# Grapevine exarcerbates the virulence of *Neofusicoccum parvum* through an overproduction of the phytotoxin terremutin

**DOI:** 10.3389/fpls.2026.1888821

**Published:** 2026-08-05

**Authors:** Idir Saber, Larissa Zeltner-Heck, Laëtitia Parent, Simon Remy, Anthony Abou Dib, Jean-Hugues Renault, Jochen Fischer-Schuch, Eckhard Thines, Florence Fontaine, Patricia Trotel-Aziz

**Affiliations:** 1 Université de Reims Champagne-Ardenne, INRAE, RIBP UMR 1488, Reims, France; 2 Institute for Biotechnology and Drug Research, Johannes Gutenberg-University, Mainz, Germany; 3 Université de Reims Champagne Ardenne, Institut de Chimie Moléculaire de Reims UMR CNRS 7312, Reims, France

**Keywords:** Botryosphaeria dieback, fungal pathogen, metabolomic, secondary metabolites, virulence factors, *Vitis vinifera*

## Abstract

**Introduction:**

Assessing the virulence of *Neofusicoccum parvum* (Np) remains challenging due to its broad host range and ability to infect numerous agriculturally and forestry important plant species. As a hemibiotrophic fungus, Np can persist as a latent endophyte before transitioning to a necrotrophic pathogen, with the timing and severity of this shift depending on strain-specific virulence. In grapevine, Np is a major causal agent of Botryosphaeria dieback, secreting phytotoxins that are strongly suspected to drive the Np lifestyle transition to necrotrophy through their tissue-necrotizing activity. However, the direct impact of a living host on Np virulence has not yet been demonstrated.

**Methods:**

In this study, we employed a sterilized grapevine plantlet model artificially inoculated with distinct Np strains to evaluate the direct incidence of a living vine on Np metabolome, including phytotoxin production. Virulence is quantified based on the percentage of necrosis in infected plantlets and correlated with metabolomic profiles specific to the Np strains. Metabolites are analyzed in both culture medium and plantlet tissues by LC-MS, with an additional HPLC quantification of the main phytotoxins mellein (M) and terremutin (T).

**Results:**

Necroses range from 11.7% with NpB-UV9 to 33.5% with Np-Bt67 at 8 dpi, then from 34.2% with NpB-UV9 to 93.3% with Np-Bt67 at 14 dpi. Phytotoxin M is consistently detected in both medium and plant tissues and is produced at high levels by strain NpB-UV9. In contrast, T is mainly detected in the culture medium and is predominantly produced by strain Np-Bt67, which caused the most rapid and extensive necrosis in plantlets. Notably, production of both toxins is stimulated by living grapevine. Additional metabolites of both fungal and plant origin are also identified.

**Discussion:**

Collectively, these results demonstrate that living grapevine exacerbates the virulence activity of distinct Np strains, for which M and T appear as conserved virulence factors relevant for detection of Np infection in necrotic grapevine tissues.

## 1 Introduction

*Neofusicoccum parvum* (Np) is a non-host specific fungus belonging to the *Botryosphaeriaceae* family, that can infect many fruits and plants of economic importance to agriculture, viticulture, and forestry (Fontaine et al., 2025; Leal et al., 2024; Mondello et al., 2018). As hemibiotroph, Np can remain latent as harmless endophyte (asymptomatic infected host) before shifting to a necrotroph pathogen leading to symptomatic infected hosts (Leal et al., 2024). Host exposure to abiotic stress could trigger the change in Np lifestyle from endophyte to necrotroph, as mentioned in the review by Leal et al. (2024).

In grapevines, the disease caused by Np is Botryosphaeria dieback (BD), a trunk disease (GTD) that causes significant economic losses in vineyards around the world (Fontaine et al., 2025; Kenfaoui et al., 2022; Mondello et al., 2018). Regardless of the intensity of this dieback, low to devastating or chronic vs apoplectic, it results in a loss of productivity, increased management costs, and a reduction in the longevity of vineyards, compromising the stability of global wine production (Gramaje and Armengol, 2011; Massonnet et al., 2018; Mugnai et al., 1999; Slippers and Wingfield, 2007; Úrbez-Torres and Gubler, 2011).

To date, no sustainable control method is currently effective enough to control BD in vineyards (Fontaine et al., 2025; Leal et al., 2024; Mesguida et al., 2023; Mondello et al., 2018), and the omnipresent pathogen spores inexorably contaminate pruning wounds and nursery baths. The highly aggressive nature of Np makes it a suitable pathogen for developing sustainable alternative control strategies against BD (Bellée et al., 2017; Larignon et al., 2015; Leal et al., 2024; Mesguida et al., 2023; Pitt et al., 2013; Úrbez-Torres and Gubler, 2011). However, the symptoms of decline induced in leaves and berries are not directly related to Np presence in these organs (Mugnai et al., 1999). Indeed, the pathogen secretes mobile phytotoxic metabolites described as virulence factors that clearly contribute to Np pathogenicity (Trotel-Aziz et al., 2019, 2022).

Studies have been conducted on the ways to successfully protect grapevines against BD using beneficial microorganisms (BCA) (Castillo-Novales et al., 2026; Leal et al., 2021; Mesguida et al., 2023; Pinto et al., 2018; Trotel-Aziz et al., 2019). The results obtained revealed the value of BCA in the indirect induced protection of vines against Np, but also their direct impacts both against Np and especially against phytotoxin accumulation. Indeed, phytotoxin direct detoxification by BCA is possible thanks to the metabolization of some and the co-metabolization of others (Trotel-Aziz et al., 2019).

In the current context of climate change, with increasing environmental constraints, plants and their BCA may struggle to achieve optimal development. It is therefore essential to also address the third determinant of plant health: the pathogen and, in particular, its virulence factors. Indeed, since GTD involve several fungi but ultimately manifest themselves as a single disease (e.g. BD), the elimination of Np could open up its ecological niche to other harmful fungi. It is therefore preferable to reduce the aggressiveness of Np rather than to kill it. To achieve this, we propose to identify what drives Np virulence and how it is exacerbated.

Among the identified phytotoxic metabolites secreted by Np, two phenolic phytotoxins have been identified: (*R*)-mellein (M) and (*T*)-terremutin (T), as well as their derivatives (Andolfi et al., 2011; Abou-Mansour et al., 2015; Evidente et al., 2010). Both are produced by polyketide synthases (PKS), as described by Trotel-Aziz et al. (2022). In necrotizing vine tissues, these secondary metabolites of phenolic nature (M and T) are strongly suspected of contributing to the expression of BD symptoms (Djoukeng et al., 2009; Abou-Mansour et al., 2015). Indeed, symptomatic diseased vines (apoplectic form) show high levels of M and T or their precursors (Magnin-Robert et al., 2016). These observations were then confirmed when searching M and T in the wood (Lemaitre-Guillier et al., 2020; Trouvelot et al., 2023) and leaves of BD-symptomatic vines (Moret et al., 2021).

The contribution of both phytotoxins to the aggressiveness of *Botryosphaeriaceae* has therefore been further characterized. The results obtained showed that M and T suppress the useful immune defenses of grapevine (i.e. JA/ET-dependent ones) and that susceptible varieties are unable to fully detoxify these phytotoxins on their own (Trotel-Aziz et al., 2019, 2022). The quantitative role of these phytotoxins in the infection process has also been demonstrated, thereby designating them as virulence factors (Trotel-Aziz et al., 2022) whose quantity is strongly suspected to force the Np lifestyle transition thanks to their necrotizing activities (Trotel-Aziz et al., 2022; Leal et al., 2024).

Diverse Np strains exist, which differ in their production of M and T *in-vitro*. The wild-type (WT) Np-Bt67 overproduces T but almost no M, the UV mutant NpB-UV9 overproduces M but almost no T, while its parent Np-B produced mid-quantity of each (Trotel-Aziz et al., 2022). When artificially inoculated *in-planta*, the Np strain producing the most severe symptoms (i.e. apoplexy) is the T overproducing (Abou-Mansour et al., 2015; Bellée et al., 2017; Trotel-Aziz et al., 2022). This reinforces the consideration on the roles of M and T metabolisms, and especially that of T, in the pathogenicity of Np. A genomic comparison has therefore been performed between the Np strains to highlight and compare the key genes involved in this differential production, in order to progress toward transformant constructions that will establish clear causal links (Trotel-Aziz et al., 2022).

Currently, despite the fact that host infection reveals a strain-dependent virulence of Np (Trotel-Aziz et al., 2022), with metabolomic signatures that include T and M (Lemaitre-Guillier et al., 2020; Magnin-Robert et al., 2016), no literature relates the living host impact on this toxin production by the Np strains upon infection. In this study, we therefore take advantage of a sterilized model of grapevine plantlets, artificially infected with distinct Np strains, to report on the direct incidence of a living vine on Np metabolome among which are phytotoxins. Virulence is assessed by the percentage of necrosis in infected plantlets in response to Np strains whose metabolome is analyzed.

Therefore, the objective is to track the metabolomic changes of Np strains in the presence or absence of the living host *Vitis vinifera* L. cv. Chardonnay, which may potentially influence the behavior of Np. Metabolites are analyzed both in culture medium and in plantlet tissues by LC-MS, with an additional HPLC analysis for the major phytotoxins mellein (M) and terremutin (T). Confirming the virulence factors of Np and their possible ways of detoxication will support later a better management of BD by improving BCA formulations with effectors that could specifically weaken Np virulence, as an essential approach in a changing environment that does not always favor the plant or its beneficial partners. Given Np’s hemibiotrophic lifestyle, which allows it to remain unnoticed for years, such metabolomic analyses will also provide markers to monitor Np aggressiveness and grapevine health directly in vineyards.

## 2 Materials and methods

### 2.1 Sterilized plant material *in-vitro* and growth conditions

Grapevine plantlets (*Vitis vinifera* L. cv. Chardonnay, clone 7535) were produced aseptically from nodal explants of 8-weeks aged plantlets. Each node was transferred onto 15 mL of agar-modified Murashige-Skoog (MS) medium in 25-mm test tubes, as described by Trotel-Aziz et al. (2008). Plantlets were maintained in a growth chamber at 25 °C day/night, with a 16h photoperiod (60 mol/m²/s).

### 2.2 Fungal strains and growth conditions

Three *Neofusicoccum parvum* (Np) strains were used in this study. The wild-type strains Np-Bourgogne S-116 (hereafter Np-B) and Np-Bt67 have been isolated respectively from French (Bourgogne area, Abou-Mansour et al., 2015) and Portuguese (Estremadura area, Reis et al., 2016) vineyards. The UV mutant of Np-B (hereafter NpB-UV9) has been produced by the University of Fribourg (Switzerland) as described in Trotel-Aziz et al. (2022). The wild-type (WT) Np-Bt67 overproduces (*-*)-terremutin (T) but almost no (*R*)-mellein (M) *in-vitro* and is described as the most aggressive *in-planta* (Abou-Mansour et al., 2015; Bellée et al., 2017; Trotel-Aziz et al., 2022). Np-B produces 3-fold less T and also produces M *in-vitro*, but is less aggressive *in-planta* than Np-Bt67 (Trotel-Aziz et al., 2022). The UV mutant NpB-UV9 overproduces M but almost no T *in-vitro*, and is as pathogenic as its parent *in-planta* (Trotel-Aziz et al., 2022).

All the Np strains were grown on potato dextrose agar medium (PDA) in darkness at 22 °C for 8 days before treatment.

### 2.3 Treatments using fungal strains

Sterilized tubes containing the MS medium without plantlets were inoculated with the distinct Np strains 8-days aged (i.e. Np-Bt67, Np-B or NpB-UV9). A plug of mycelial agar (5 mm diameter) was aseptically deposited on the MS medium. Control consisted of non-inoculated tubes.

Sterilized tubes containing 6-weeks aged plantlets were also inoculated with a mycelial plug of Np-Bt67, Np-B or NpB-UV9. The plug was aseptically deposited on the MS medium near the plantlet stem. Control consisted of non-inoculated tube that contained a plantlet.

Each treatment lasted two weeks under the condition of the growth chamber (25 °C day/night, with a 16h photoperiod at 60 mol/m²/s). Each experiment was replicated four times, with three samples per replicate.

### 2.4 Phenotyping and sampling

Two time periods were considered both for phenotyping and sampling: 8 and 14 days post-inoculation (dpi).

Phenotyping corresponds to the percentage of necrosis per plantlet (due to Np), obtained by the ratio between the necrotic length of each plantlet and the total length of the plantlet.

For sampling, both the culture medium and the plantlet tissues were collected. For one replicate per condition: 3 culture media were transferred into a 50 mL Falcon tube, and 3 plantlets were collected into a 15 mL Falcon tube. All tubes were immediately immersed in liquid nitrogen to preserve sample integrity, then stored at -80 °C until further analyses.

### 2.5 Extraction of the metabolome from Np-infected culture medium and Np-infected plantlets

From Np-infected culture medium, metabolome was extracted using a standardized procedure. To each tube, 20 mL methanol (MeOH) were added, followed by an orbital agitation (24 °C, 16 h). The extracts were filtered through Whatman filter paper using a vacuum pump. An aliquot of approximately 1.5 mL of the filtrate was transferred into sterile, labeled HPLC vials for further analyses. The same was done with non-infected culture medium.

From Np-infected plantlets, extraction was also performed with pure MeOH to obtain an extract concentration of 100 mg/mL. The mixture was then subjected to orbital agitation at 24 °C for approximately 16 h, before filtering through Whatman paper under vacuum. An aliquot of about 1.5 mL of the filtrate was transferred into sterile, labeled HPLC vials for further analyses. The same was done with non-infected plantlets.

### 2.6 UPLC-MS/MS analysis and data preprocessing

Metabolomic analysis by UPLC-MS/MS was directly performed from the HPLC vials containing the metabolome from: (i) Np-infected culture medium without plantlets, (ii) Np-infected culture medium with plantlets, (iii) non-infected culture medium, (iv) Np-infected plantlets, and (v) non-infected plantlets.

Chromatographic analyses were conducted on a Thermo Scientific UPLC 3000 system coupled to an Orbitrap mass spectrometer equipped with a heated electrospray ionization (HESI) source. System control and data acquisition were performed using Xcalibur™ software (Thermo Fisher Scientific). Separation was achieved on a C18-HQ analytical column (150 mm × 2.1 mm, 2.2 µm; Interchim, France) maintained at 40 °C. The mobile phase consisted of solvent A (water with 0.1% formic acid) and solvent B (acetonitrile with 0.1% formic acid). A binary gradient elution was applied at a flow rate of 0.4 mL min^-^¹: starting at 5% B, linearly increasing to 100% B from 0 to 19 min, held isocratically at 100% B for 6 min, and then re-equilibrated to 5% B for 5 min. The injection volume was 4 µL, and the concentration of the analyzed extracts was 5 mg mL^-^¹.

Mass spectrometric detection was performed in positive ionization mode. The HESI parameters were as follows: spray voltage 3.46 kV, sheath gas 49 a.u., auxiliary gas 20 a.u., and sweep gas 1 a.u. The capillary temperature and auxiliary gas heater were set to 321 °C and 303 °C, respectively. The S-lens RF level was 55. Full MS spectra were acquired over m/z 100–1500 at a resolution of 35,000 FWHM (m/z 200), with an AGC target of 1 × 10^6^ and a maximum injection time of 100 ms. Data-dependent MS² (dd-MS²) acquisition was performed in Top3 mode at a resolution of 17,500 FWHM, using an AGC target of 1 × 10^5^ and a maximum injection time of 50 ms. The three most intense precursor ions from each full MS scan were isolated with a 1.0 Da window and fragmented using stepped normalized collision energies of 10, 30, and 50 units. Dynamic exclusion was applied with a 3 s exclusion time, and isotope exclusion was enabled to minimize redundant fragmentation.

Data preprocessing was performed using MZmine 4.7.29 (https://github.com/mzmine/mzmine/releases), while statistical analyses were carried out through MetaboAnalyst 6.0, an online platform dedicated to metabolomics data analysis. Normalization was conducted in R software to minimize experimental bias and enhance comparability across samples. Specifically, data were normalized by the median, followed by cube root transformation and autoscaling. After normalization, multivariate analyses such as PCA, PLS-DA, and VIP score evaluation were applied to explore metabolic variations and identify discriminant metabolites between experimental groups. The VIP score is a commonly used metric in PLS-based models to estimate the contribution of each variable to the model, with variables showing VIP > 1 generally considered as the most influential for class discrimination (Chong and Jun, 2005; Wold et al., 2001). Metabolite networks were also constructed using MZmine 4.7.29 and visualized with Cytoscape 3.10.3 to facilitate the exploration of molecular relationships.

The culture medium alone (no Np nor plantlet) was used as a blank control. This condition was excluded from multivariate analyses (PLS-DA and VIP scoring) in order to avoid clustering due to metabolites derived from the culture medium rather than to biologically relevant variation. Nevertheless, it was retained in the molecular network to allow the annotation and identification of clusters corresponding to medium-derived compounds.

### 2.7 HPLC-UV analysis

HPLC analysis was also directly performed from the HPLC vials, to quantify the two targeted phytotoxins (e.g. terremutin and mellein) and to validate the LC-MS analysis tracking these two molecules.

Samples were analyzed using an Ultimate 3000 Dual-Gradient HPLC (DIONEX) coupled to a photodiode array detector (PDA). Chromatographic conditions were as follows: Acclaim™ 120 C18 column (150 × 4.6 mm, 5 µm; Thermo Scientific), using isocratic mobile phase with water:acetonitrile (H_2_O:ACN) 50:50 v/v containing 0.1% phosphoric acid (H_3_PO_4_). Detection was recorded at 210 and 273 nm for (*R*)-mellein and (-)- terremutin, respectively. Phytotoxin identification was confirmed by UV spectrum and retention time: (*R*)-mellein was eluted with 1 mL/min of ACN:H2O 60:40 v/v at 3.8 min, while (*-*)-terremutin was eluted with 0.7 mL/min of ACN:H2O 10:90 v/v at 5.8 min. Concentration was determined using standard curves as described by Trotel-Aziz et al. (2019, 2022).

### 2.8 Statistical analysis

Statistical analyses of HPLC-UV data were performed to assess the significance of differences in (*R*)-mellein and (*-*)-terremutin production between samples with or without the plant. A *z*-test (equivalent to a *t*-test with known variances) was applied to directly compare two conditions, using the formula: z = M1 - M2/√(SEM1^2^ + SEM2^2^) where M1​ and M2​ are the means, and SEM1 and SEM2​ are the standard errors of the mean. Analyses were carried out using RStudio (version 2024.09.0 + 375).

The HPLC/MS-MS intensity data for (*R*)-mellein and (-)-terremutin, and the other VIPs were analyzed using non-parametric statistics in R (v4.3.3). Because the data did not meet normality and homoscedasticity assumptions, a Kruskal-Wallis test was performed to compare intensity distributions among biological classes. When significant, *post-hoc* pairwise comparisons were conducted using Dunn’s test with Bonferroni correction. Boxplots were generated using ggplot2 and ggpubr, showing log10-transformed intensities with significant differences indicated by asterisks (*p < 0.05, **p < 0.01, ***p < 0.001, ****p < 0.0001).

## 3 Results

### 3.1 Aggressiveness of Np strains toward grapevine plantlets in sterilized conditions

**Figure 1:**
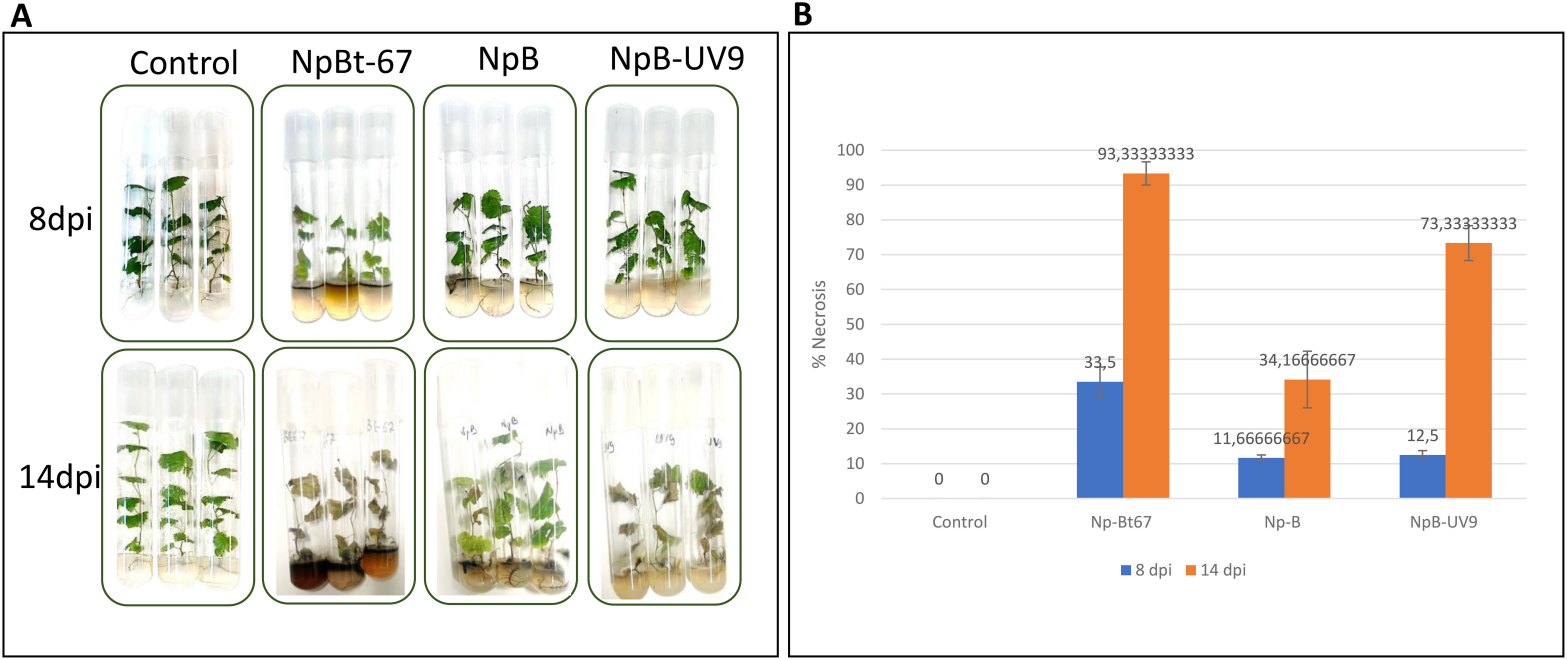
Aggressiveness of the *N. parvum* strains (Np-Bt67, Np-B, and NpB-UV9) toward grapevine plantlets in sterilized conditions at 8 and 14 days post-inoculation (dpi). **(A)** Visual phenotyping of the *in-vitro* Chardonnay infected plantlets. **(B)** Percentage of necrosis in plantlets at 8 dpi (blue) and 14 dpi (orange).

As shown in **Figure 1**, the percentage of necrosis in plantlets is the highest when challenged with Np-Bt67. At 8 dpi, this percentage reached 33.5% in plantlets challenged with Np-Bt67, while it reached 11.7% and 12.5% in presence of Np-B and NpB-UV9 respectively. Similarly, at 14 dpi, Np-Bt67 remains the most aggressive toward plantlets showing a necrosis of 93.3%, compared to plantlets confronted with Np-B (34.2% necrosis) and NpB-UV9 (73.3% necrosis). Over the same period, the progression of aggressiveness of Np-B is the most moderate (11.7% to 34.2%) compared to NpB-UV9 (12.5% to 73.3%) and Np-Bt67 (33.5% to 93.3%) the most aggressive (**Figure 1**).

### 3.2 Metabolomic analysis of Np-infected culture medium

#### 3.2.1 Molecular network

**Figure 2:**
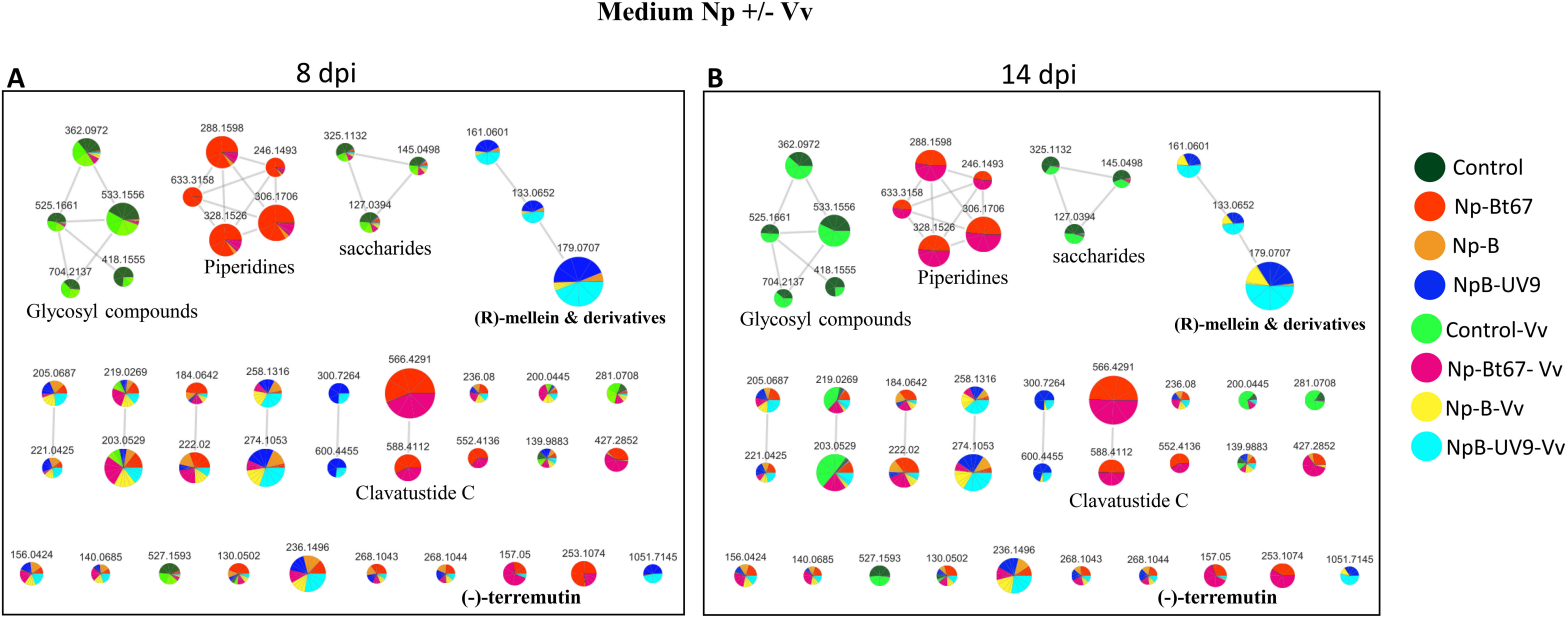
Molecular networks constructed from LC-MS/MS data from *N. parvum* culture medium (Np-Bt67, Np-B and NpB-UV9) at 8 dpi **(A)** and 14 dpi **(B)**, whether plantlets are absent or present (Vv). Node size is proportional to compound intensity, and color distribution within each node indicates the relative proportion of intensity associated with each experimental modality. Related annotation is in **Table 1**.

In Np-infected culture medium, whether plantlets are absent or present, some nodes appear most intense both at 8 and 14 dpi, with specific colors corresponding to specific experimental conditions (**Figure 2**).

Among them, a cluster corresponds to (*R*)-mellein and derivatives. In this cluster, the most intense node (i.e. 179.0707 m/z) is annotated as the precursor ion of (*R*)-mellein. The two associated fragments with lower intensities correspond to the ions [M–H_2_O+H]^+^ and [M–CH_2_CHO+H]^+^ (i.e. 161.0601 m/z and 133.0652 m/z, respectively) that confirm the typical structure of (*R*)-mellein. The color distribution within this cluster indicates that the majority of (*R*)-mellein and derivatives are produced by the strain NpB-UV9, both at 8 and 14 dpi, and whether plantlets were absent or present. In a weaker extent, (*R*)-mellein and derivatives are also produced by Np-B. Interestingly, at 14 dpi, (*R*)-mellein and derivatives appear highly produced in the Np-infected culture medium when plantlets are present. In contrast, the ion corresponding to the chemically fragile (*-*)-terremutin appears isolated in the network, with no connection to other nodes. However, the color distribution shows that (*-*)-terremutin is mainly produced by Np-Bt67 and is also significantly enhanced with the plantlet.

Other major clusters have also been annotated (**Figure 2**; **Table 1**). Two clusters highlight molecules that are overexpressed in the non-infected control medium, with or without the plantlets (i.e. glycosylated compounds and saccharides). Two other clusters highlight metabolites that are almost exclusively produced by Np-Bt67 (i.e. piperidines and derivatives, and Clavatustide C), but whether the plantlets are absent or present.

**Table 1:** Annotation of the metabolites associated with the clusters identified by an untargeted metabolomic molecular network approach on extracts of the culture medium of *Neofusicoccum parvum* strains (Np-Bt67, Np-B and NpB-UV9) in the presence or absence of plantlets, using SIRIUS v6.2.2.

clusters	metabolites							
	n°	M/Z	RT	Adduct	Formula	MW	Candidate structures	Class
Piperidines	1	306.17	12.41	[M+H]^+^	C_17_H_23_NO_4_	305.4	1-[(6-Ethyl-1-oxo-2,3,3a,6,7,7a-hexahydroindene-4-carbonyl)amino]-2-methylcyclopropane-1-carboxylic acid	Piperidine
	2	328.15	12.41	[M+Na]^+^	C_17_H_23_NO_4_	305.4	1-[(6-Ethyl-1-oxo-2,3,3a,6,7,7a-hexahydroindene-4-carbonyl)amino]-2-methylcyclopropane-1-carboxylic acid	Piperidine
	3	288.16	12.41	[M-H_2_O+H]^+^	C_17_H_23_NO_5_	305.4	1-[(6-Ethyl-1-oxo-2,3,3a,6,7,7a-hexahydroindene-4-carbonyl)amino]-2-methylcyclopropane-1-carboxylic acid	Piperidine
	4	633.32	12.41	[2M+Na]^+^	C_17_H_23_NO_6_	305.4	1-[(6-Ethyl-1-oxo-2,3,3a,6,7,7a-hexahydroindene-4-carbonyl)amino]-2-methylcyclopropane-1-carboxylic acid	Piperidine
	5	246.15	12.39	[M-C_2_H_4_O_2_+H]^+^	C_17_H_23_NO_7_	305.4	1-[(6-Ethyl-1-oxo-2,3,3a,6,7,7a-hexahydroindene-4-carbonyl)amino]-2-methylcyclopropane-1-carboxylic acid	Piperidine
glycosides polaires	6	533.15	1.2	[M+H]^+^	C_21_H_24_N_8_O_7_S	532.5	5'-O-[(L-Tryptophylamino)sulfonyl]adenosine	Glycosylamines
	7	362.09	1.19	[M-C_5_H_9_N_5_S+H]^+^	C_16_H_15_N_3_O_7_	361	5'-O-[(L-Tryptophylamino)sulfonyl]adenosine fragment	
	8	525.16	1.19	[M+H_2_O+H]^+^	C_17_H_30_O_17_	506.4	Cellobiosylglucose	Oligosaccharides
	9	704.21	1.19				uknown	
	10	418.15	1.2	[M+H_2_O+H]^+^	C_14_H_25_NO_12_	399.35	[(3S,4S,6R)-3,4,5-trihydroxy-6-[(2R,3R,5S)-3,4,5-trihydroxy-6-(hydroxymethyl)oxan-2-yl]oxyoxan-2-yl]methyl 2-aminoacetate	Glycosylamines
Mellein & derivatives	11	179.07	10.75	[M+H]^+^	C_10_H_10_O_3_	178.18	Mellein	Phenols
	12	133.06	10.74	[M+H]^+^	C_9_H_8_O	132	Mellein fragment	Phenols
	13	161.06	10.75	[M-H_2_O+H]^+^	C_10_H_10_O_3_	178.18	Mellein	Phenols
Sugar-derived compounds	14	325.11	1.25	[M+H]^+^	C_12_H_20_O_10_	324.28	Lactosan	Disaccharides
	15	145.05	1.26	[M+H]^+^	C_9_H_8_O_4_	144.12	5-(1,2-dihydroxyethyl)-3(2H)-furanone	Furanones
	16	127.04	1.25	[M-H_2_O+H]^+^	C_9_H_8_O_4_	144.12	5-(1,2-dihydroxyethyl)-3(2H)-furanone	Furanones
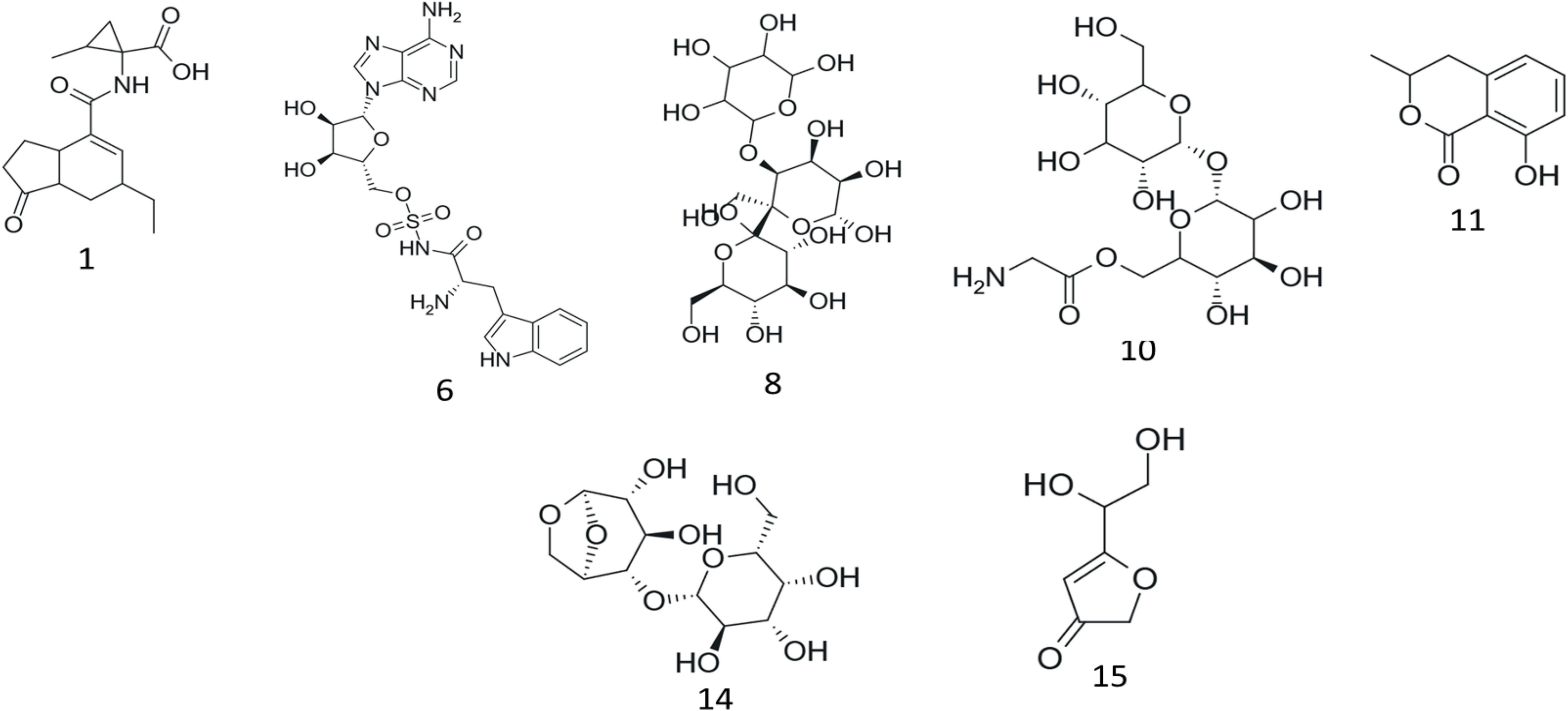								

#### 3.2.2 Comparative analysis over time of each Np strain culture medium

##### 3.2.2.1 Metabolites discriminating over time in each Np culture medium

**Figure 3:**
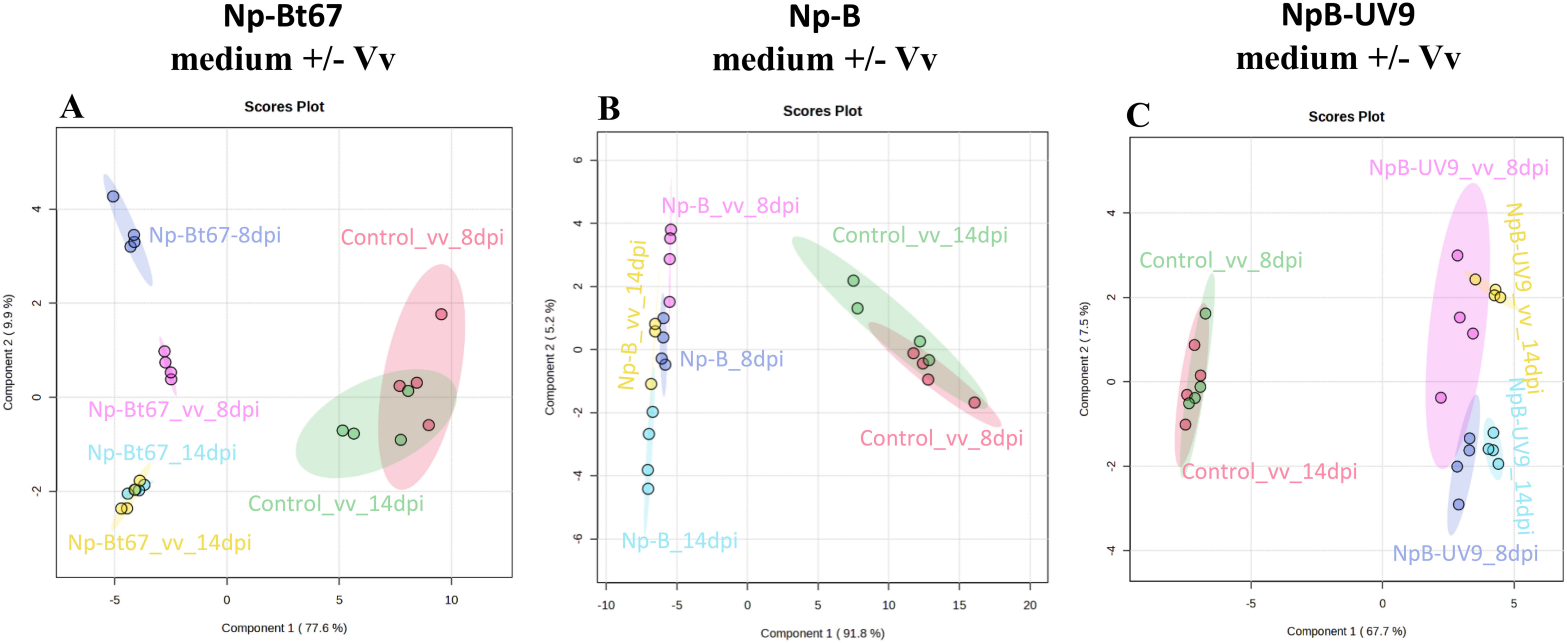
PLS-DA analyses illustrating metabolic discrimination over time in each *N. parvum* culture medium at 8 dpi (t1) and 14 dpi (t2), whether plantlets are absent or present (Vv). The Np strains are Np-Bt67 **(A)**, Np-B **(B)** or NpB-UV9 **(C)**.

The Np metabolome is first analyzed for each strain to distinguish any possible specific metabolite, whether plantlets are absent or present (**Figure 3**).

For strain Np-Bt67, the PLS-DA score plot shows a clear separation between experimental groups along the selected two principal components that cover 87.5% of the variability of the data (**Figure 3A**). The confidence ellipses highlight good intra-group consistency (low dispersion of replicates) and a distinct separation between control and infected samples. A marked temporal effect appears in Np-Bt67 culture medium between 8 and 14 dpi, while the ellipses overlap in the non-infected control with plantlets. A plantlet effect is also observed at 8 dpi in the Np-Bt67 culture medium, compared to the same Np-Bt67 culture medium without plantlets. Cross-validation and permutation testing confirm the robustness of the model. Accuracy increases from 0.15 (1 component) to 0.88 (4 components), reflecting a strong classification ability. The determination coefficients (R²) range from 0.49 to 0.67, indicating a good explanation of the variance, while the predictive values (Q²) between 0.23 and 0.40 indicate a moderate but acceptable predictive power (Hair et al., 2016; Henseler et al., 2009). The permutation test (p < 0.001) confirms the statistical significance of the model, excluding random separation. Overall, despite a moderate Q², these results demonstrate the presence of a specific metabolic signature associated with Np-Bt67 infection, post-infection dynamics, and plantlet presence.

For strain Np-B, the score plot shows a good separation between experimental groups along the selected two principal components that explain 97.0% of the total variance (**Figure 3B**). Clear metabolic differences discriminate between infected samples and controls. In the Np-B infected conditions, additional metabolic differences related to time and plantlet presence are also observed. Cross-validation results show a progressive improvement in model performance with the number of components: Accuracy: from 0.17 (1 component) to 0.62 (3–4 components), reflecting a satisfactory classification ability; R²: from 0.47 to 0.54, indicating a good proportion of explained variance; Q²: from 0.38 to 0.44, suggesting a better predictive power than that observed for Np-Bt67. The permutation test (p = 0.001) confirms the statistical significance of the model and rules out random separation between groups.

Overall, these results indicate that infection by Np-B induces a specific and significant metabolic response, with a clear separation between experimental conditions and statistical stability supported by cross-validation and permutation testing.

The PLS-DA analysis for NpB-UV9 also shows a very strong discrimination between experimental groups with the selected two principal components that cover 75.2% of the total variance (**Figure 3C**). As observed with the other Np strains: samples from the NpB-UV9 culture medium clearly separate from controls and discriminate over time (8 and 14 dpi) to contrast with the non-infected control with plantlet. An additional plantlet effect is also observed in the NpB-UV9 culture medium (i.e. NpB-UV9 vs NpB-UV9_Vv) both at 8 and 14 dpi. Cross-validation results indicate excellent model performance: Accuracy: from 0.25 (1 component) to 0.78 (4 components), showing a strong classification capacity; R²: from 0.71 to 0.98, demonstrating a high proportion of explained variance; Q²: from 0.64 to 0.90, indicating a high predictive power and excellent model stability. Finally, the permutation test (p < 0.001) confirms the statistical significance of the model, showing that the observed separation is not random.

In brief: (i) the PLS-DA obtained are robust, predictive, and statistically significant, confirming the biological relevance of the observed results; and (ii) results indicate that (a) the metabolic changes occurring in the Np culture medium are related to Np metabolome, and that (b) plantlet would influence the metabolic responses of Np, unless they mutually influence the content of their culture medium. Focus needs now to be done to identify any specificity due to the Np strains, influenced both by time and by the presence of the host plant.

##### 3.2.2.2 Identity of the main metabolites discriminating over time in each Np culture medium

**Figure 4:**
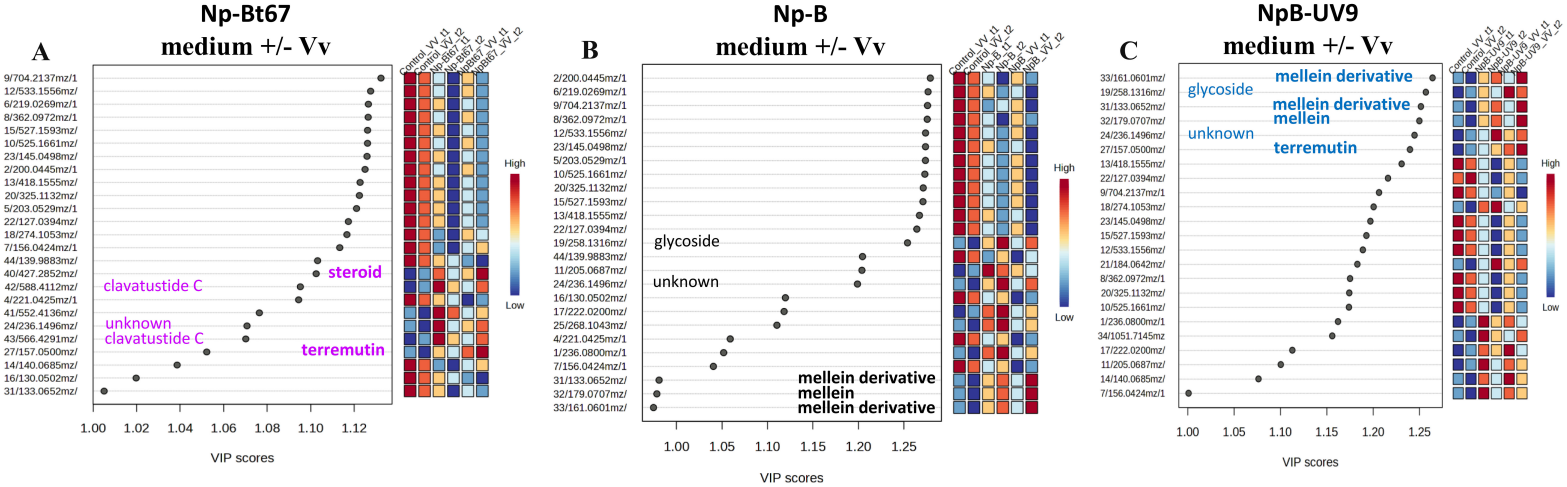
Score plots displaying the top VIPs (Variable Importance in Projection) metabolites in each *N. parvum* culture medium at 8 dpi (t1) and 14 dpi (t2), whether plantlets are absent or present (Vv). Only metabolites with VIP values greater than 1 are shown. The color gradient from blue to red on the right indicates the relative accumulation of each metabolite across conditions: dark red denotes overaccumulation, while blue indicates less/no accumulation. The Np strains are Np-Bt67 **(A)**, Np-B **(B)** or NpB-UV9 **(C)**. Related annotation is in **Table 2**.

To identify the metabolites contributing most to the discrimination observed in the previously analyzed PLS-DA, the VIP (Variable Importance in Projection) scores have been calculated for each Np culture medium (Np-Bt67, Np-B, and NpB-UV9) whether plantlets are absent or present (**Figure 4**). These scores reflect the relative importance of each variable in the construction of the model and enable the compounds most involved in the differentiation between the experimental groups to be determined. Variables with a VIP score greater than 1 (VIP > 1) have been selected to discriminate metabolites. The related annotation performed using SIRIUS 6.2.2 software is detailed in **Table 2**.

**Table 2:** SIRIUS v6.2.2 annotations of the most discriminant metabolites over time (8 and 14 dpi) in each *N. parvum* culture medium, whether plantlets are absent or present (VV).

n°	VIP	Vipscore Np-Bt67	Vipscore Np-B	Vipscore NpB-UV9	Adduct	MW	Formula	Candidate structure	Class	Sirius Score	Tanimoto score
1	704.2137mz/1.19min	1.1324	1.276	1.2064	uknown						
2	533.1556mz/1.20min	1.1276	1.2738	1.1889	[M+H]^+^	491.5	C_21_H_24_N_8_O_7_S	5'-O-[(L-Tryptophylamino)sulfonyl]adenosine	Glycosylamines	0.9999	0.5075
3	219.0269mz/1.19min	1.1266	1.2766	ns	[M+Na]^+^	196.16	C_9_H_8_O_5_	2,6-Diacetyl-3-hydroxypyran-4-one	Heteroaromatic compounds	0.9875	0.5
4	362.0972mz/1.19min	1.1265	1.2757	1.1751	uknown						
5	527.1593mz/1.21min	1.1262	1.271	1.1927	[M+Na]^+^	504.4	C_18_H_32_O_16_	Galactosucrose	Oligosaccharides	0.9998	0.8198
6	525.1661mz/1.19min	1.1262	1.2731	1.1739	[M+H2O+H]^+^	506.4	C_17_H_30_O_17_	Cellobiosylglucose	Oligosaccharides	0.3333	0.8787
7	145.0498mz/1.26min	1.1259	1.2738	1.197	[M+H]+	144.12	C_6_H_8_O_4_	5-hydroxy-4-methoxy-5,6-dihydro-2H-pyran-2-one	Furanones	1	0.6181
8	200.0445mz/1.16min	1.125	1.2791	ns	uknown						
9	418.1555mz/1.20min	1.1228	1.2671	1.2307							
10	325.1132mz/1.25min	1.1224	1.2713	1.1742	[M+H]+	324.28	C_12_H_20_O_10_	Lactosan	Disaccharides	0.9996	0.7961
11	203.0529mz/1.19min	1.1211	1.2737	ns	uknown						
12	127.0394mz/1.25min	1.1174	1.2642	1.216	[M+H]^+^	144.12	C_9_H_8_O_4_	4H-Pyran-4-one, 5-hydroxy-2-methyl-	Pyranones	0.0172	0.5652
13	274.1053mz/1.23min	1.1167	ns	1.2005	[M+K]^+^	235.28	C10H21NO5	4-aminobutyl alpha-L-fucopyranoside	glycoside	0.9993	0.2
14	156.0424mz/1.19min	1.1134	1.0406	1.0009	[M+K]**^+^**	117.15	C_5_H_11_NO_2_	Betaine	Alpha amino acids and derivatives	0.8934	0.7292
15	139.9883mz/26.76min	1.1032	1.2045	ns	uknown						
16	427.2852mz/14.28min	1.1026	ns	ns	[M+H2O+H]^+^	408.266	C27H36O3	[(E)-3-[(3S,10R,13S)-3-acetyloxy-10,13-dimethyl-2,3,4,7,8,9,11,12,14,15-decahydro-1H-cyclopenta[a]phenanthren-17-yl]but-2-enyl] acetate	Steroid esters	1	0.6173
17	588.4112mz/15.09min	1.0953	ns	ns	[M+Na]^+^	565.8	C_30_H_55_N_5_O_5_	Clavatustide C	Cyclopeptide	0.9999	0.73099
18	221.0425mz/1.18min	1.0945	1.0591	ns	uknown						
19	552.4136mz/14.49min	1.0764	ns	ns	[M+H]^+^	551.8	C_29_H_53_N_5_O_5_	Luminmide B	Cyclopeptide	1	0.875
20	236.1496mz/1.35min	1.0708	1.199	1.2446	uknown						
21	566.4291mz/15.09min	1.0702	ns	ns	[M+H]^+^	565.8	C_30_H_55_N_5_O_5_	Clavatustide C	Cyclopeptide	1	0.8431
22	157.0500mz/3.28min	1.0523	ns	1.2397	[M+H]^+^	156.14	C_7_H_8_O_4_	Terremutin	coumarin		
23	140.0685mz/1.21min	1.0386	ns	1.0763	[M+Na]^+^	117.15	C_5_H_11_NO_2_	Betaine	Amino acids and derivatives	1	0.7757
24	130.0502mz/1.23min	1.0198	1.1198	ns	[M+H]^+^	129.04	C_5_H_7_NO_3_	DL-Pyroglutamic acid	Proline and derivatives	1	0.8814
25	133.0652mz/10.74min	1.0051	ns	1.2515	[M+H]^+^	132	C_9_H_8_O	Mellein fragment	Mellein derivative		
26	258.1316mz/1.25min	ns	1.254	1.2568	[M+Na]^+^	235.28	C_10_H_21_NO_5_	4-aminobutyl alpha-L-fucopyranoside	glycoside	0.9598	0.18239
27	205.0687mz/1.20min	ns	1.2039	1.1004	[M+H]+	204.14	C_4_H_8_N_6_O_4_	1,1-Ethylenebis(1-nitrosourea)	Alpha amino acids	0.01668	0.5769
28	268.1043mz/1.35min	ns	1.1104	ns	[M+H]^+^	267.24	C_10_H_13_N_5_O_4_	Adenosine	Purine nucleosides	1	0.9826
29	236.0800mz/1.12min	ns	1.0519	1.1621	uknown						
30	161.0601mz/10.75min	ns	ns	1.2641	[M-H2O+H]+	178.18	C_10_H_10_O_3_	Mellein	Phenols	0.9999	0.69608
31	179.0707mz/10.75min	ns	ns	1.2498	[M+H]^+^	178.18	C_10_H_10_O_3_	Mellein	Phenols	0.9998	0.7378
32	184.0642mz/1.25min	ns	ns	1.183	uknown						
33	1051.7145mz/10.96min	ns	ns	1.1559	uknown						
34	222.0200mz/1.23min	ns	1.1183	1.1128	uknown						
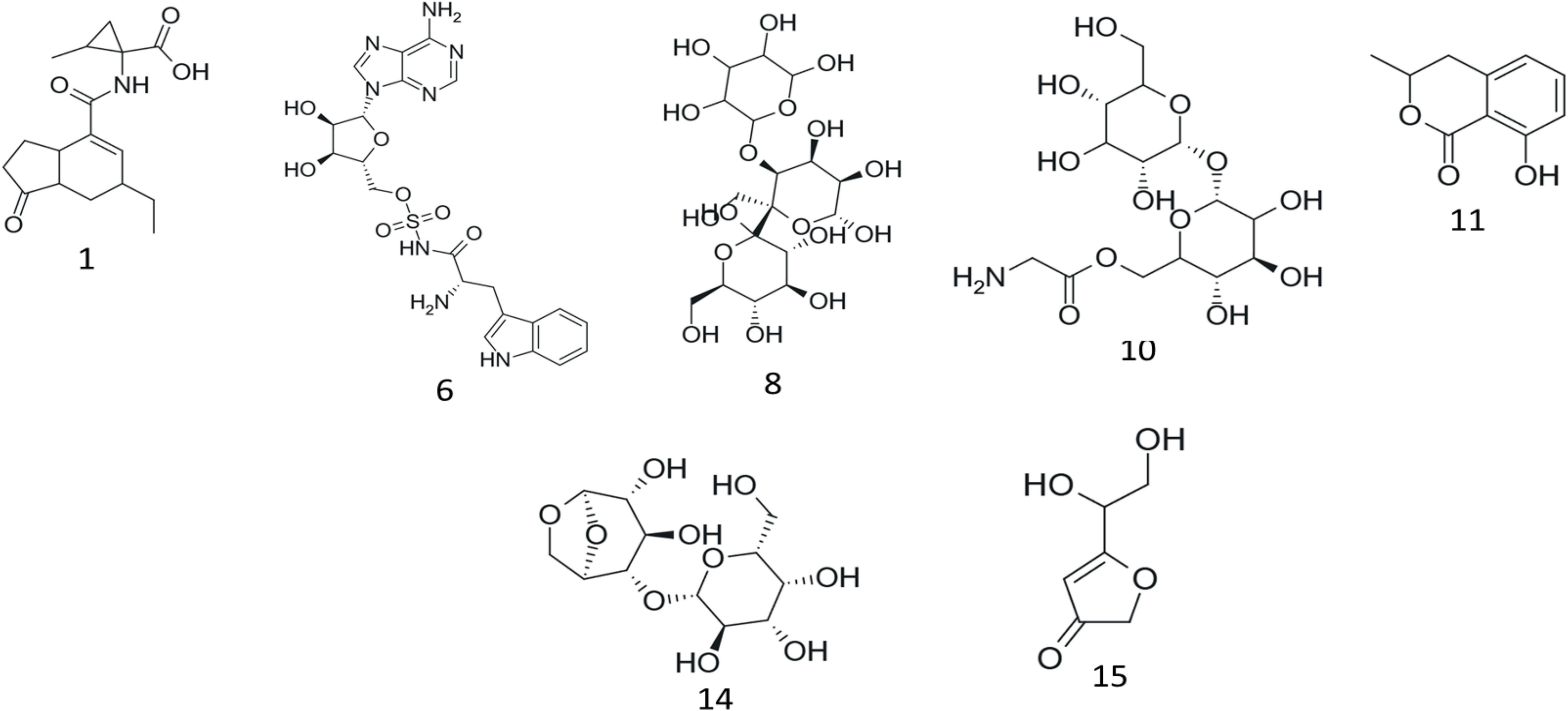											

Data include: SIRIUS scores (confidence of molecular formula assignment) and Tanimoto similarity values (structural similarity between predicted and candidate molecules). Color code for common discriminant metabolites to : **blank**, the three strains; **grey**, Np-Bt67 and Np-B; **orange**, Np-Bt67 and NpB-UV9; **green**, NpB and NpB-UV9; or specific metabolites to : **red**, Np-Bt67; **blue**, Np-B; **yellow**, NpB-UV9.

Among these metabolites, (-)-terremutin is annotated as highly produced by Np-Bt67 metabolome in the presence of the plantlet, with an even more marked accumulation at 14 dpi (**Figure 4A**, **Table 2**). This metabolite has a VIP score greater than 1, indicating a significant contribution to the discrimination observed in the PLS-DA model. Another metabolite follows the same trend (i.e. a steroid ester), as part of Np-Bt67 metabolome in the presence of the plantlet. Clavatustide C also discriminates at 14 dpi in the culture medium of Np-Bt67 when plantlet is present, but in a weaker intensity than to (-)-terremutin (**Figure 4A**).

Similarly, (*R*)-mellein and derivatives highly discriminate in the metabolome of NpB-UV9 particularly at 14 dpi with the plantlet (**Figure 4C**; **Table 2**). The (*-*)-terremutin also discriminates in NpB-UV9 culture medium when plantlet is present, with a more marked accumulation at 14 dpi.

These two toxins, (*R*)-mellein and (*-*)-terremutin also discriminate in the Np-B metabolome in the presence of the plantlet, but with VIP values below 1 that reflect a lesser contribution to group separation for this model (**Figure 4B**, **Table 2**).

Interestingly, a glycoside also appears as a discriminant metabolite in the culture medium of the less aggressive Np-B, with and without plantlets, at 14 dpi (**Figure 4B**) and in the NpB-UV9 culture medium with plantlets both at 8 and 14 dpi (**Figure 4C**).

Other discriminant metabolites are also associated with Np metabolome, showing a VIP score higher than 1 (**Figure 4**; **Table 2**). However, despite that most of these metabolites have been successfully annotated, almost all of them discriminate less when the plantlet is present.

#### 3.2.3 Comparative analysis between the Np-strains

##### 3.2.3.1 Metabolites discriminating by comparing the Np strain’s culture medium

**Figure 5:**
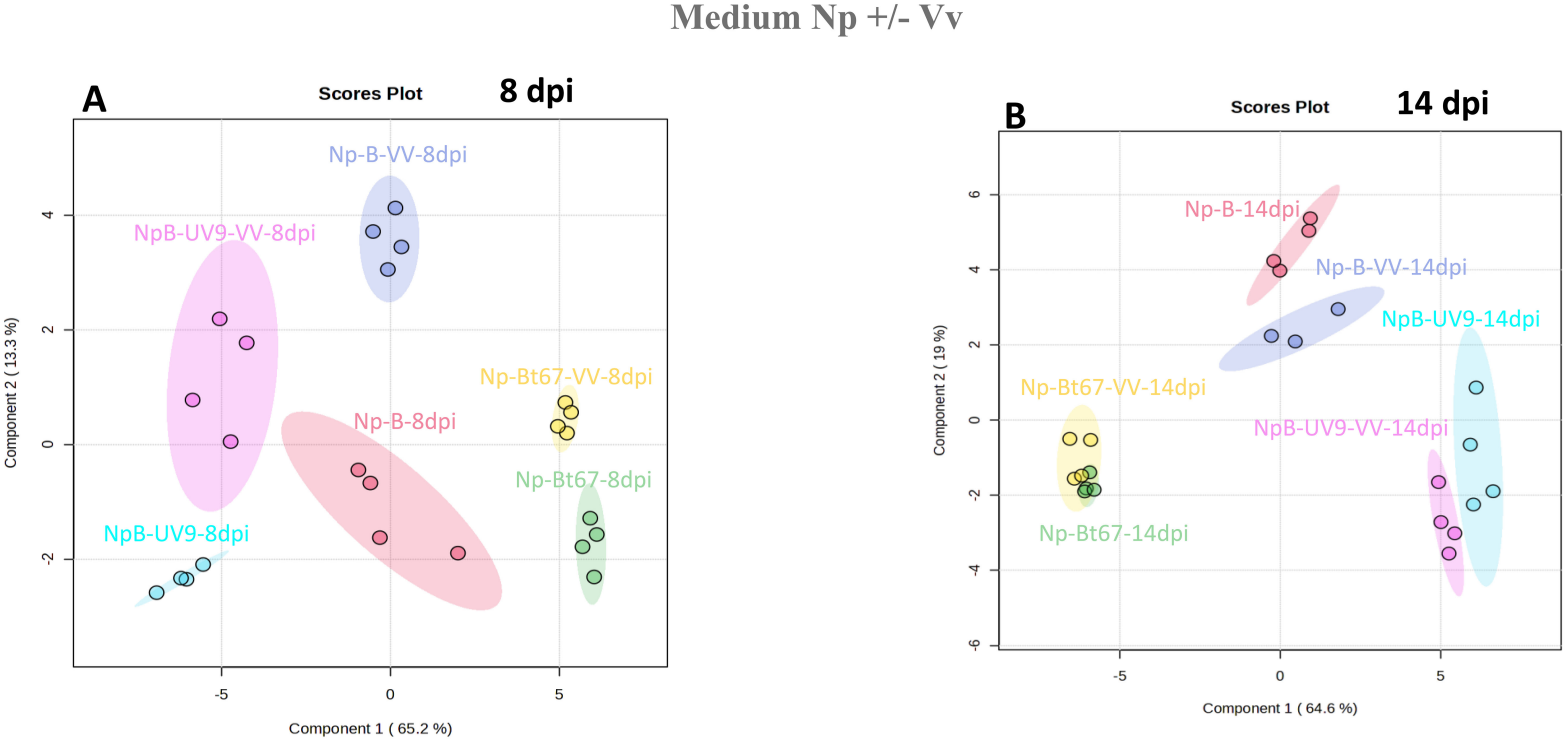
PLS-DA analyses illustrating metabolic discrimination by comparing the Np strain’s culture medium (Np-Bt67, Np-B and NpB-UV9) at 8 dpi **(A)** and 14 dpi **(B)**, whether plantlets are absent or present (Vv).

PLS-DA analysis was performed to compare the metabolic profiles of the culture medium of the three Np strains (**Figure 5**).

At 8 dpi, the selected two principal components cover 78.5% of the total variance and enable a clear separation between the Np strains, with a marked discrimination of NpB-UV9 from Np-B and Np-Bt67 (**Figure 5A**). The robustness of the model is supported by cross-validation, showing an improvement in predictive ability as the number of components increases (Q²> 0.4 starting from 3 components), as well as by the permutation test (p < 0.001), confirming that the observed discrimination is not due to chance.

Similar results are observed at 14 dpi when the selected two principal components cover 83.6% of the total variance and clearly separate the three Np strains (**Figure 5B**). Cross-validation indicates good predictive performance (Q² > 0.5 for 2–3 components), and the permutation test (p < 0.001) confirms the statistical validity of the model.

Overall, these analyses demonstrate that each strain exhibits a specific metabolic signature that is reproducible over time.

##### 3.2.3.2 Identity of the main metabolites discriminating by comparing the Np strain’s culture medium

**Figure 6:**
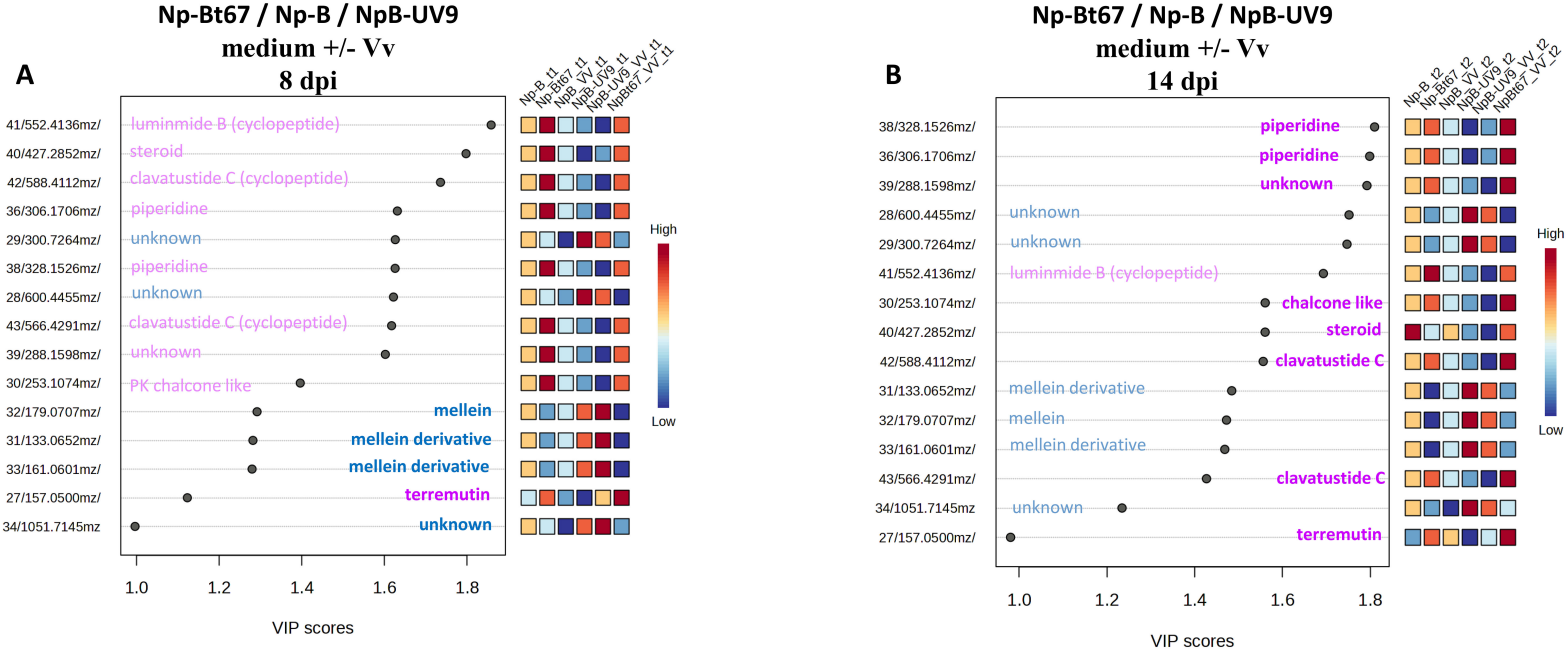
Score plots displaying the top VIPs (Variable Importance in Projection) metabolites discriminating by comparing the Np strain’s culture medium (Np-Bt67, Np-B and NpB-UV9) either at 8 dpi (A, t1) and 14 dpi (B, t2), whether plantlets are absent or present (Vv). Only metabolites with VIP values greater than 1 are shown. The color gradient from red to blue on the right indicates the relative expression levels of each metabolite across conditions: red denotes overaccumulation, while blue indicates less/no accumulation. Bold metabolites (right side) are those over increased by Vv. Related annotation is detailed in **Supplementary Table 3**.

The variables that are important for prediction (VIP) have been selected based on a VIP score > 1 (**Figure 6**) and then annotated, which made it possible to identify the metabolites contributing to the discrimination between the three strains (**Supplementary Table 3**). As reported with the previous analyses (**Figures 2**, **4**): (-)-terremutin highly discriminates with Np-Bt67 metabolome and (*R*)-mellein and derivatives highly discriminate in the metabolome of NpB-UV9, both at 8 dpi (**Figure 6A**) and 14 dpi (**Figure 6B**). Mellein and derivatives over-accumulate in Np-culture medium in the presence of the plantlet, particularly at 8dpi (**Figure 6A**). Terremutin over-accumulates in Np-culture medium in the presence of the plantlet at both time periods (**Figures 6A, B**). Interestingly, in the metabolome of Np-Bt67: a piperidine, two cyclopeptides (i.e. clavatustide C, luminmide B), a steroid ester and a polyketide (PK) chalcone like also discriminate in Np-Bt67 metabolome and over-accumulate in the Np-culture medium in the presence of the plantlet (**Figure 6**; **Supplementary Table 3**).

#### 3.2.4 Differential levels of the metabolites identified as discriminating in each Np culture medium

Three metabolites commonly discriminate in the analyses based on time comparison (**Figure 4**) and on Np comparison (**Figure 6**): (*R*)-mellein, (*-*)-terremutin and a steroid. Based on their already described aggressiveness, only (*R*)-mellein and (*-*)-terremutin have been retained to apprehend their differential level.

**Figure 7:**
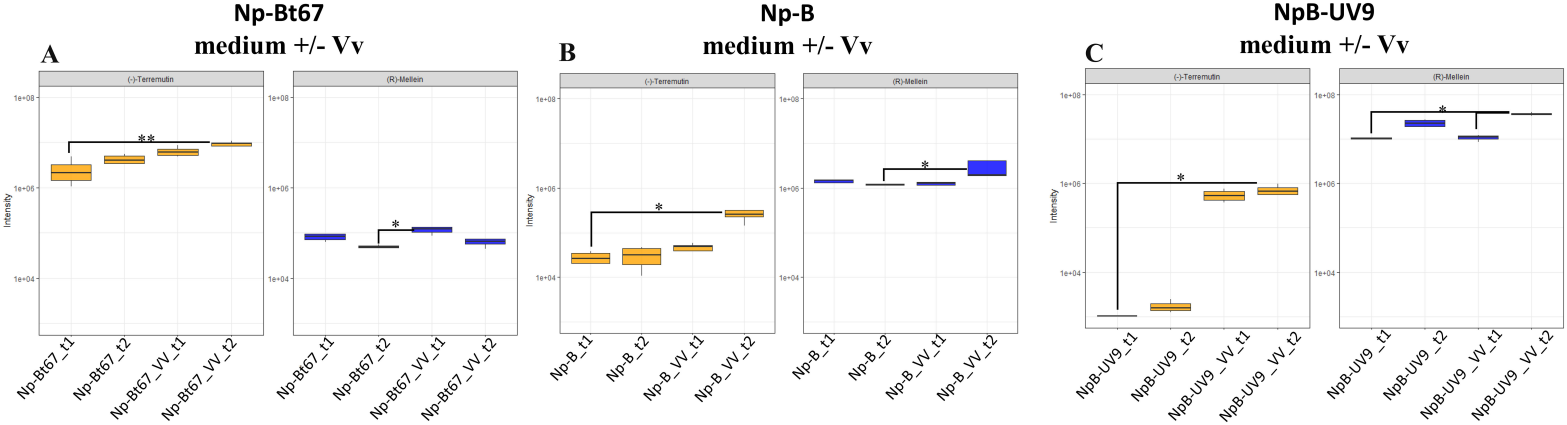
Variations in LC–MS intensities of (*R*)-mellein and (*-*)-terremutin illustrating their differential levels in each *N. parvum* culture medium at 8 dpi (t1) and 14 dpi (t2) whether plantlets are absent or present (Vv). The Np strains are Np-Bt67 **(A)**, Np-B **(B)** or NpB-UV9 **(C)**. Neither (*R*)-mellein nor (*−*)-terremutin were detected in non-infected culture medium or in plantlets alone. The Kruskal–Wallis test revealed significant differences for both toxins. *Post hoc* pairwise comparisons were performed using Dunn’s test with Bonferroni correction, and significant differences are indicated by asterisks (*p < 0.05, **p < 0.01, ***p < 0.001).

The relative abundances of (*R*)-mellein (M) and (-)-terremutin (T) in the Np culture medium according to the time post-infection and the presence of the host plantlet are shown in the **Figure 7**. Similarly to the previous analysis, Np-Bt67 overproduces T (**Figure 7A**), while NpB-UV9 overproduces M (**Figure 7C**) and Np-B produces intermediate quantities of M and T (**Figure 7B**). The presence of plantlet in the Np culture medium stimulates the production of M and T by the three Np strains, particularly at 14 dpi, with statistical analyses showing significant differences between infected samples without plantlet and infected samples with plantlet. Interestingly, while T is almost absent from the metabolome of NpB-UV9, but T production appears with the plantlet presence, highlighting a host-dependent production of T production by NpB-UV9.

In brief: these results highlight a marked influence of plantlet on toxin biosynthesis by the Np strains, with distinct production dynamics depending on the strain and the time post-infection.

### 3.3 Metabolomic analysis of Np-infected plantlets

The pre-processing of data carried out using MZmine 4.7.29 allowed only 13 metabolites of usable quality to be retained (**Table 3**), a significantly lower number than that obtained during the analysis of the culture medium, reflecting the complexity and lower intensity of metabolic signals in the plantlet matrix. The extraction procedure, targeting fungal secondary metabolites and phytotoxins, may also contribute to this relatively low number of metabolites detected in plant tissues. The same statistical approaches as for culture medium have been applied to the plantlets.

**Table 3:** SIRIUS v6.2.2 annotation of the most discriminant metabolites identified by PLS-DA in *Vitis vinifera* plantlets when comparing Control plantlets to Np-Infected plantlets over time (Np-Bt67, Np-B and NpB-UV9).

n°	VIP	Vipscore Np-Bt67	Vipscore Np-B	Vipscore NpB-UV9	Adduct	MW	Formula	Candidate structure	Class	Sirius Score	Tanimoto score
1	147,077mz/1.18min	1.2669	1.119	1.2048	[M+H_3_N+H]^+^	129.11	C_5_H_7_NO_3_	Pyroglutamic Acid	amino acid derivative	100%	90.43%
2	130,050mz/1.18min	1.2697	1.1196	1.2088	[M+H]^+^	129.11	C_5_H_7_NO_3_	Pyroglutamic Acid	amino acid derivative	100%	85.95%
3	156,42mz/1.19min	ns	ns	1.0131	[M+K]^+^	117.16	C_5_H_11_NO_2_	Betaine	amino acid derivative	99.50%	60.42%
4	116,071mz/1.22min	ns	1.0268	ns	Unkown						
5	161,060mz/5,67min	ns	1.224	1.249	[M-H2O+H]+	178.18	C_10_H_10_O_3_	(R)-Mellein derivative	Phenols	1.04%	58.10%
6	179,071mz/5,69min	ns	1.2025	1.2429	[M+H]^+^	178.18	C_10_H_10_O_3_	(R)-Mellein	Phenols	100%	75.73%
7	363,106mz/5,70min	ns	1.2287	1.246	[M+Na]^+^	340.11	C_16_H_20_O_8_	Mellein-8-O-β-D-glucopyranoside	Phenol derivatives		
8	289,071mz/7,32min	1.2895	1.0437	1.009	[M+H]^+^	288.25	C_15_H_12_O_6_	Eriodictyol	Flavanones	100%	98.91%
9	179,071mz/10,75min	ns	1.1698	1.1402	[M+H]^+^	178.18	C_10_H_10_O_3_	(R)-Mellein	Phenols	100%	79.62%
10	566,429mz/15,09min	1.3096	ns	ns	[M+H]^+^	565.8	C_30_H_55_N_5_O_5_	Clavatustide C	Cyclopeptide	100%	83.76%
11	588,411mz/15,10min	1.3096	ns	ns	[M+Na]^+^	565.8	C_30_H_55_N_5_O_5_	Clavatustide C	Cyclopeptide	100%	63.05%
12	621,272mz/19,36min	ns	1.0583	1.0297	[M+H]^+^	620.7	C_36_H_36_N_4_O_6_	Methyl phaeophorbide b	chlorophyll derivative	100%	63.16%
13	607,293mz/20,63min	1.2896	1.1305	1.1673	[M+H]^+^	606.7	C_36_H_38_N_4_O_5_	Methyl pheophorbide a	chlorophyll derivative	99.88%	80.33%
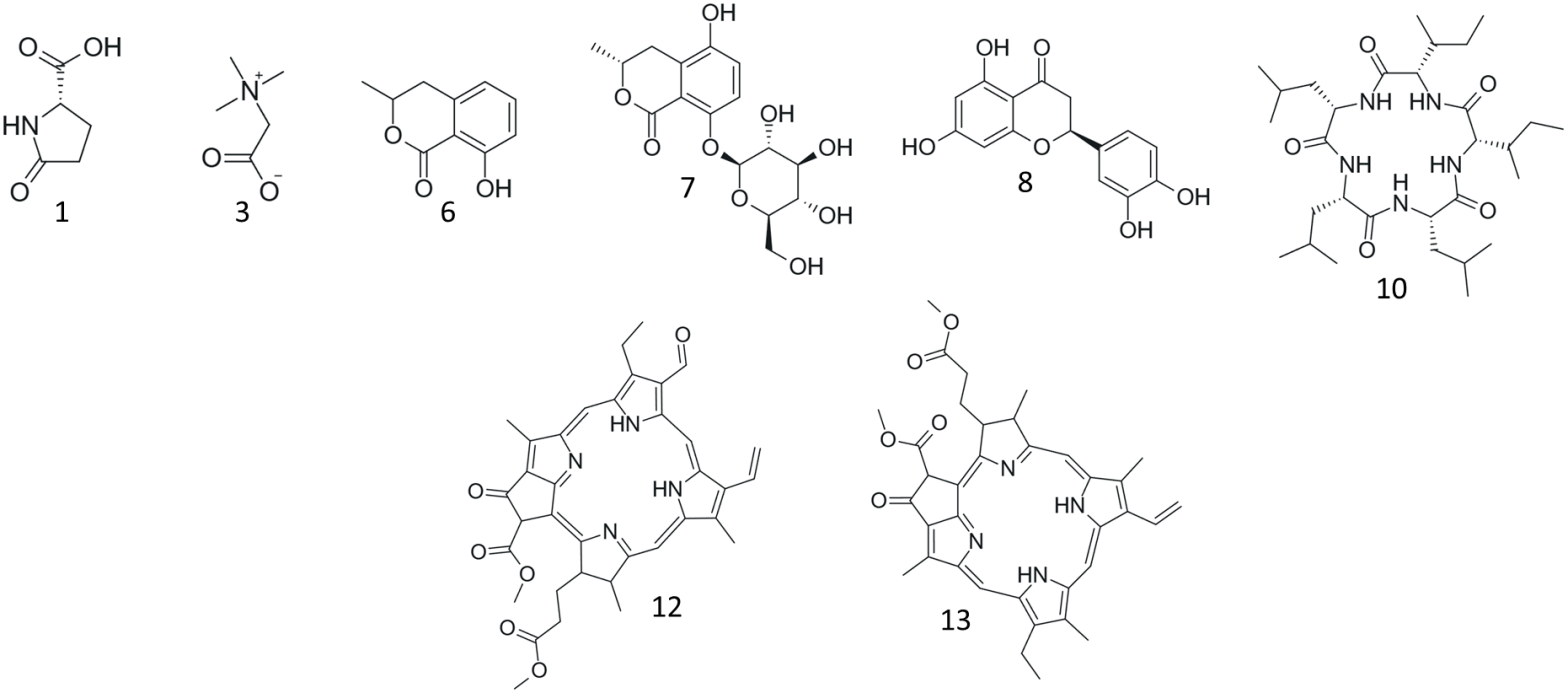											

Discrimination is therefore based on time for each Np strain. Data include: SIRIUS scores (confidence of molecular formula assignment) and Tanimoto similarity values (structural similarity between predicted and candidate molecules) based on time. Color code for common discriminant metabolites to : **blank**, the three strains ; **green**, NpB and NpB-UV9; or specific metabolites to : **red**, Np-Bt67; **blue**, Np-B; **yellow**, NpB-UV9.

#### 3.3.1 Molecular network

**Figure 8:**
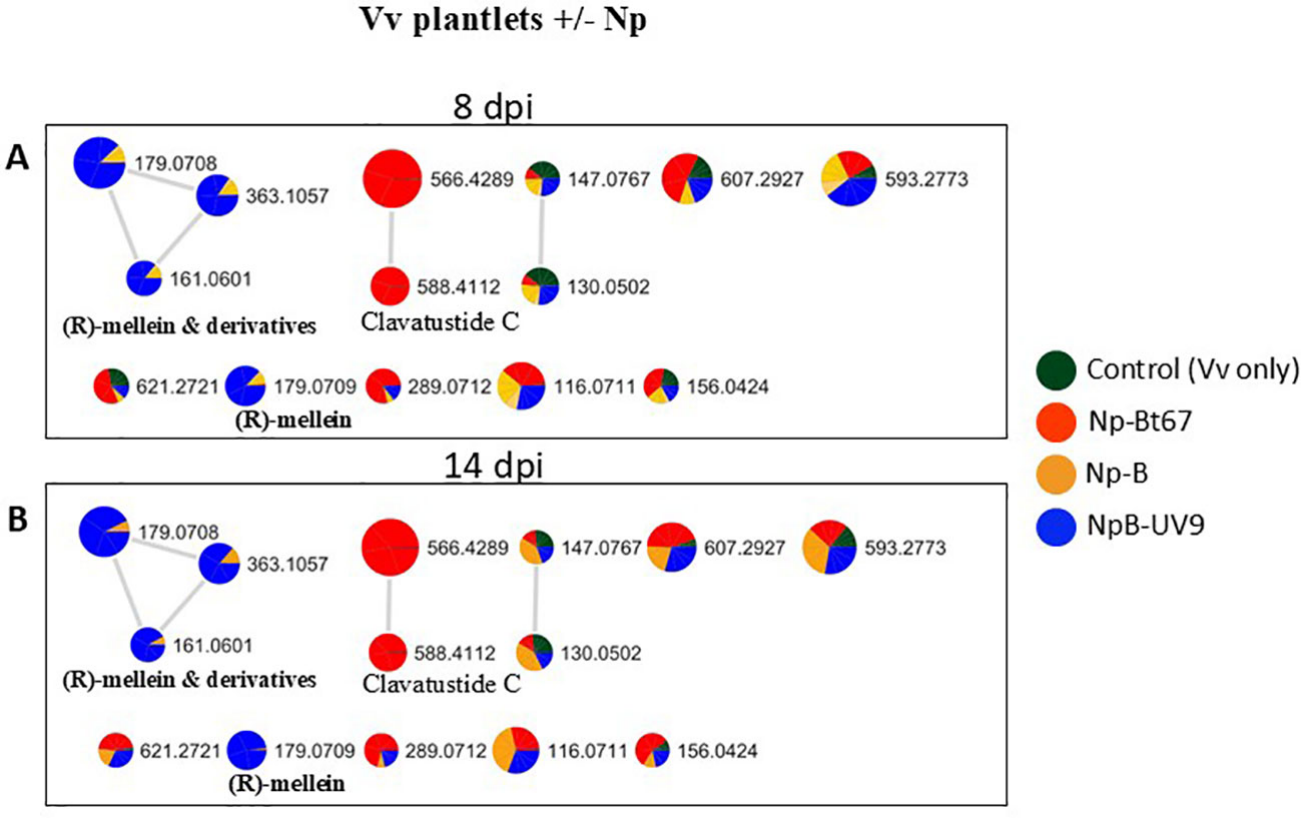
Molecular networks constructed from LC–MS/MS data from grapevine plantlets infected by the *N. parvum* strains (Np-Bt67, Np-B and NpB-UV9) or not (Control), both at 8 dpi **(A)** and 14 dpi **(B)**. Node size is proportional to compound intensity, and color distribution within each node indicates the relative proportion of intensity associated with each experimental modality. Related annotation is in **Table 3**.

As shown in **Figure 8**, the molecular network generated by MzMine 4.7.29 revealed three main small clusters in Np-infected plantlets. The first is the (*R*)-mellein cluster (precursor ion at 179.07 m/z), linked to its fragment [M+H–H_2_O]^+^ at 161.06 m/z and its glycosylated derivative, mellein-O-glucoside [M+Na]^+^ at 363.11 m/z.

The other two clusters are binary: one that groups clavatustide C [M+H]^+^ at 566.43 m/z, linked to its sodium adduct [M+Na]^+^ at 588.41 m/z; the other combines pyroglutamic acid [M+H]^+^ at 130.05 m/z with an ion at 147.07 m/z corresponding to the ammonium form [M+H_3_N+H]^+^ (**Figure 8**).

In line with the previous data, (*R*)-mellein and derivatives highly discriminate in plantlets infected by NpB-UV9, while clavatustide C highly discriminates in plantlets infected by Np-Bt67. As already annotated in the Np-infected culture medium, the absorption of these molecules by plantlets is not excluded.

#### 3.3.2 Comparative analysis of the Np-infected plantlets based on time

##### 3.3.2.1 Metabolites discrimination over time

**Figure 9:**
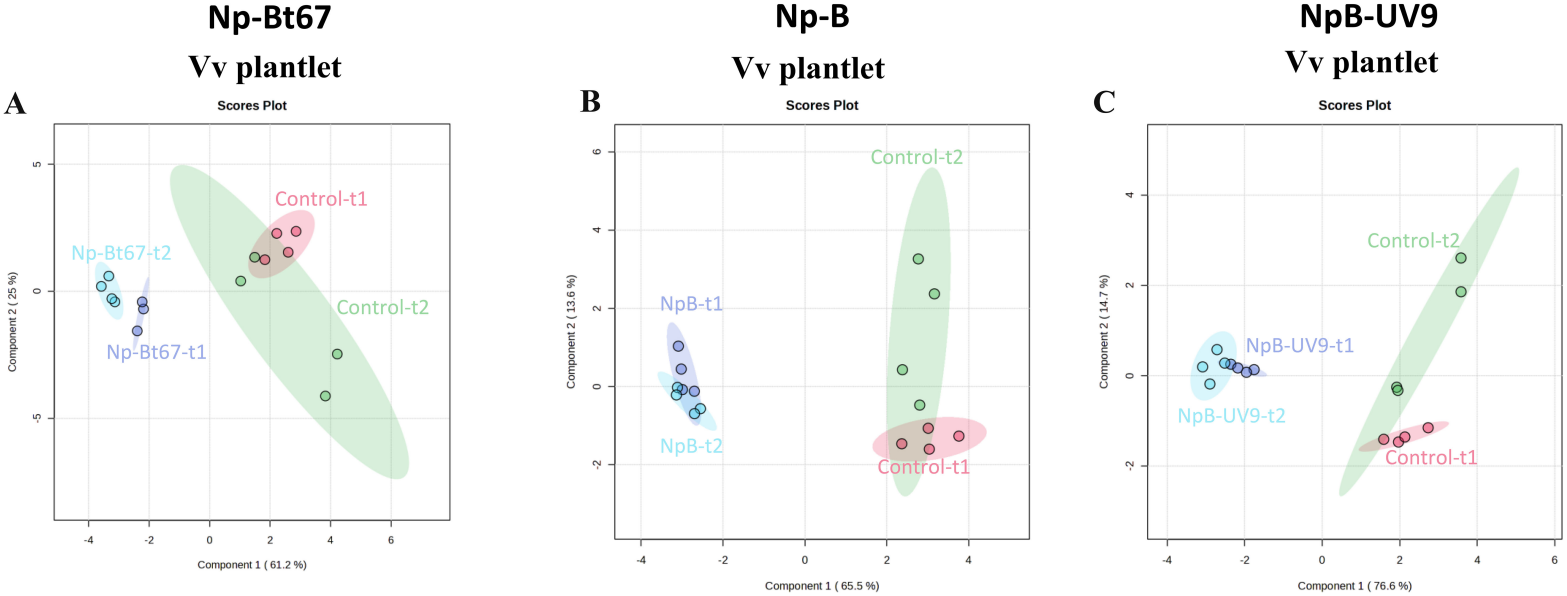
PLS-DA models showing the discrimination of metabolic profiles of plantlets infected with the *N. parvum* strains Np-Bt67 **(A)**, Np-B **(B)** or NpB-UV9 **(C)** or not (Control), both at 8 dpi (t1) and 14 dpi (t2). Related annotation is in **Table 3**.

As shown in **Figure 9**, the PLS-DA analyses are performed by comparing the Np-infected plantlets to non-infected plantlets (control) over time, with the distinct Np strains (Np-Bt67, Np-B, or NpB-UV9) considered separately.

In plantlets infected by Np-Bt67 (**Figure 9A**), PLS-DA shows a clear separation between Np-infected plantlets and control plantlets, explained by the first two principal components (Comp1: 61.2%; Comp2: 25%). Intra-group variability appears low, indicating the good homogeneity of replicates, except for the control plantlets at 14 dpi showing a greater dispersion. Cross-validation (Accuracy: 0.4 → 0.76; Q²: 0.01 → 0.70) and permutation testing (p < 0.001) confirm the robustness and significance of the PLS-DA model, with good classification ability and high predictive power from the second component onwards.

In plantlets infected by Np-B (**Figure 9B**), PLS-DA also shows good separation between Np-infected plantlets and control plantlets (Comp1: 65.5%; Comp2: 13.6%). A low intra-group variability is also observed in the control plantlets at 14 dpi. However, the metabolomic differences between the Np-infected plantlets at 8 dpi and 14 dpi appear less contrasted upon Np-B infection (**Figure 9B**) than with Np-Bt67 (**Figure 9A**). Cross-validation (Accuracy: 0.30 → 0.60; Q²: 0.25 → 0.53) and the permutation test (p < 0.001) confirm the consistency and statistical significance of the model.

In plantlets infected by NpB-UV9 (**Figure 9C**), the first two components explain a large part of the variance (Comp1: 76.6%; Comp2: 14.7%). A clear separation is also observed between Np-infected plantlets and control plantlets, and the control plantlets at 14 dpi also show intra-group variability. However, the metabolomic differences between the NpB-UV9-infected plantlets at 8 dpi and 14 dpi appear less contrasted (**Figure 9C**) than with Np-B (**Figure 9B**). Cross-validation (Accuracy: 0.37 → 0.80; Q²: -0.05 → 0.62) and the permutation test (p < 0.001) indicate excellent performance and robustness of the PLS-DA model.

Overall, these results show that Np induces detectable metabolic changes in the plantlets present in the same culture medium, despite a limited number of metabolites retained after pretreatment.

##### 3.3.2.2 Identity of the main discriminating metabolites over time

**Figure 10:**
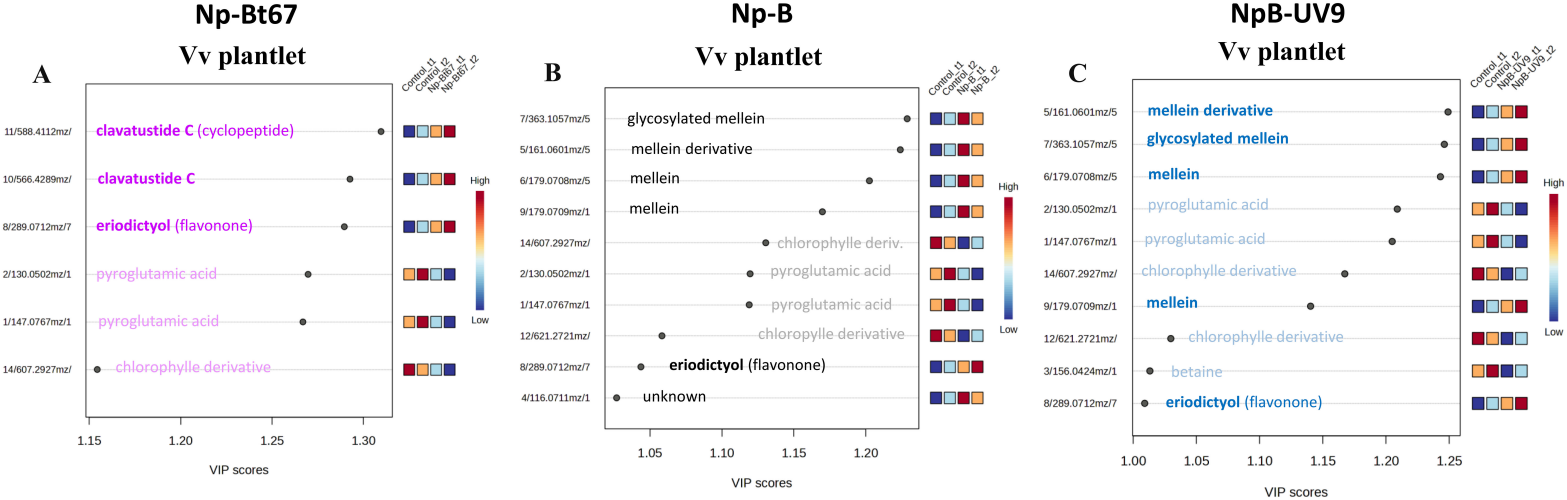
Score plots displaying the top VIPs (Variable Importance in Projection) metabolites in plantlets infected with the *N. parvum* strain Np-Bt67 **(A)**, Np-B **(B)** or NpB-UV9 **(C)** or not (Control), both at 8 dpi (t1) and 14 dpi (t2). Metabolites shown have VIP values greater than 1. The color gradient from red to blue on the right indicates the relative expression levels of each metabolite across conditions: red denotes overaccumulation, while blue indicates less/no accumulation. Bold metabolites are those over increased *in-planta* over time. Related annotation is detailed in **Table 3**.

The metabolites discriminating the most between modalities were identified based on the VIP values of the PLS-DA models (**Figure 10**). Only metabolites with a VIP score greater than 1 were selected annotations (**Figure 10**, **Table 3**).

When the comparative analysis is based on time, eriodictyol (C_15_H_12_O_6_) appears as a common discriminant metabolite in infected plantlets whatever the Np strain (**Figure 10**). Upon Np-Bt67 challenge, the cyclopeptide clavatustide C (C_30_H_55_N_5_O_5_) discriminate highly in plantlets, particularly at 14 dpi (**Figure 10A**). In plantlets infected by Np-B and NpB-UV9, (*R*)-mellein and derivatives highly discriminate at 8 dpi with Np-B (**Figure 10B**) and 14 dpi with NpB-UV9 (**Figure 10C**).

In non-infected plantlets (Control), other metabolites discriminate such as chlorophyll derivatives, pyroglutamic acid and betaine.

#### 3.3.3 Comparative analysis of Np-infected plantlets based on the Np strains

##### 3.3.3.1 Metabolites discrimination based on the Np strains

**Figure 11:**
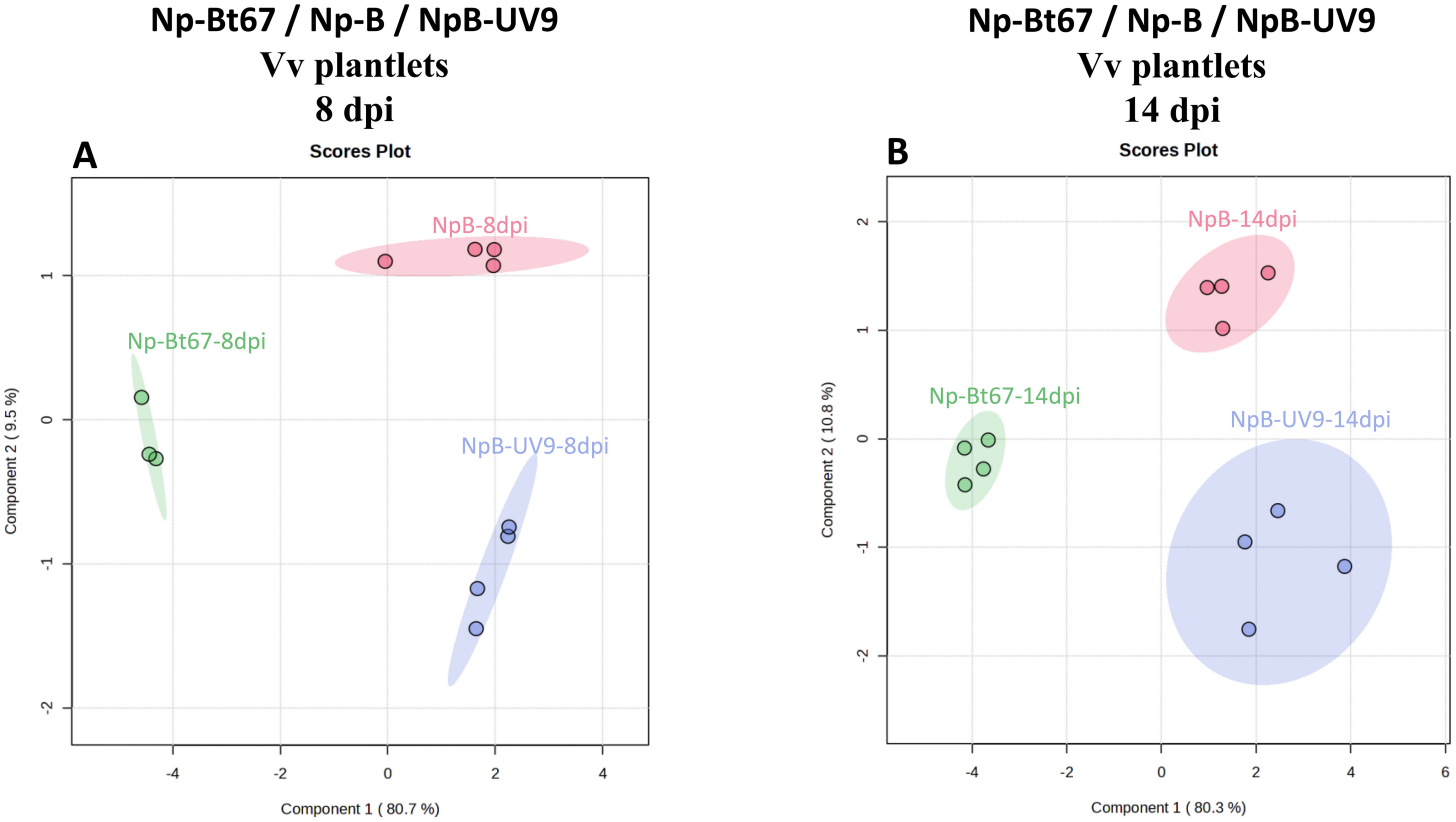
PLS-DA analyses showing the discrimination of metabolic profiles of plantlets infected with the Np strains Np-Bt67, Np-B or NpB-UV9 at 8dpi **(A)** and 14 dpi **(B)**.

As shown in **Figure 11**, the PLS-DA analyses compare the metabolic profiles of plantlets infected by Np-Bt67, Np-B or NpB-UV9 both at 8 dpi (**Figure 11A**) and 14 dpi (**Figure 11B**).

At 8 dpi (Comp1: 80.7%; Comp2: 9.5%), the model shows a clear separation between the Np strains, with a marked discrimination of Np-Bt67 from Np-B and NpB-UV9 along the first component, while the second component contributes to a finer distinction between the groups (**Figure 11A**). The robustness of the model is confirmed by cross-validation, with high performance (Q² > 0.8 starting from 2 components), as well as by the permutation test (p = 0.032), indicating that the observed discrimination is statistically significant.

At 14 dpi (Comp1: 80.3%; Comp2: 10.8%), the three Np strains remain clearly separated, with a similar group structure (**Figure 11B**). Cross-validation also shows good predictive capabilities (Q² > 0.7 for 2–3 components), and the permutation test (p = 0.005) confirms the model’s robustness.

##### 3.2.3.2 Identity of the main discriminating metabolites

**Figure 12:**
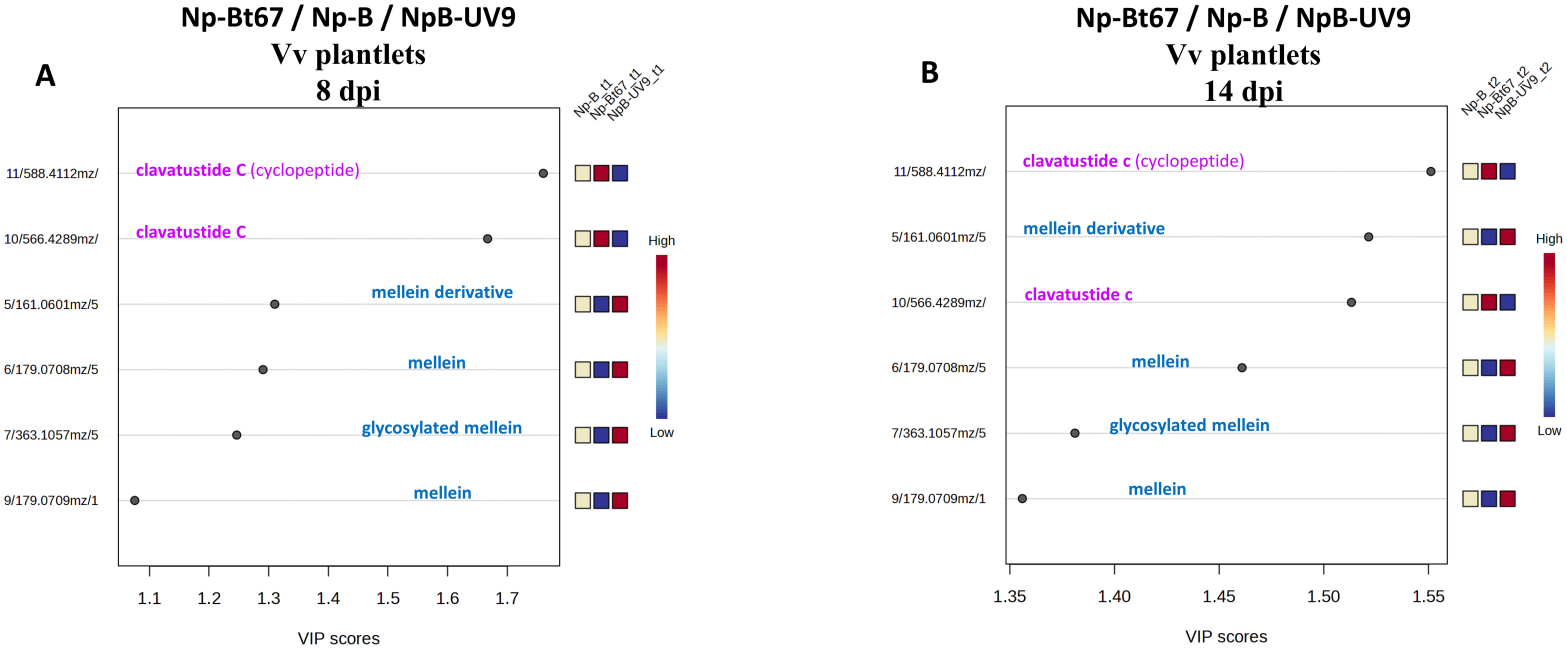
Score plots displaying the top VIPs (Variable Importance in Projection) metabolites discriminating in plantlets infected by the *N parvum* strains (Np-Bt67, Np-B or NpB-UV9) at 8 dpi **(A)** and 14 dpi **(B)**. These metabolites VIP values are greater than 1. The color gradient from red to blue on the right indicates the relative expression levels of each metabolite across conditions: red denotes overaccumulation, while blue indicates less/no accumulation. Related annotation is in **Supplementary Table 5**.

The variables important for projection (VIP) were selected based on a VIP score > 1 (**Figure 12**) and then annotated (**Supplementary Table 5**) to identify the main discriminating metabolites in Np-infected plantlets based on the Np-strain comparison.

As shown in **Figure 12**, the cyclopeptide clavatustide C (C_30_H_55_N_5_O_5_) discriminates highly in plantlets infected by Np-Bt67 and the (*R*)-mellein group discriminates in plantlets infected by NpB-UV9, both at 8 dpi (**Figure 12A**) and 14 dpi (**Figure 12B**).

Overall (**Schema 1**), these results confirm a specific metabolic signature in Np-infected plantlets, depending on the specific metabolome of the Np strains (**Figure 4**) from which some metabolites can directly integrate the plantlet tissues (**Figure 10**, i.e. clavatustide C and mellein with derivatives) to contrast with (*-*)-terremutin that is too hydrophilic, while others metabolites could directly be produced by the plantlets (i.e. the flavonone eriodictyol).

#### 3.3.4 Differential levels of the main metabolites identified as discriminating in infected plantlets

**Figure 13:**
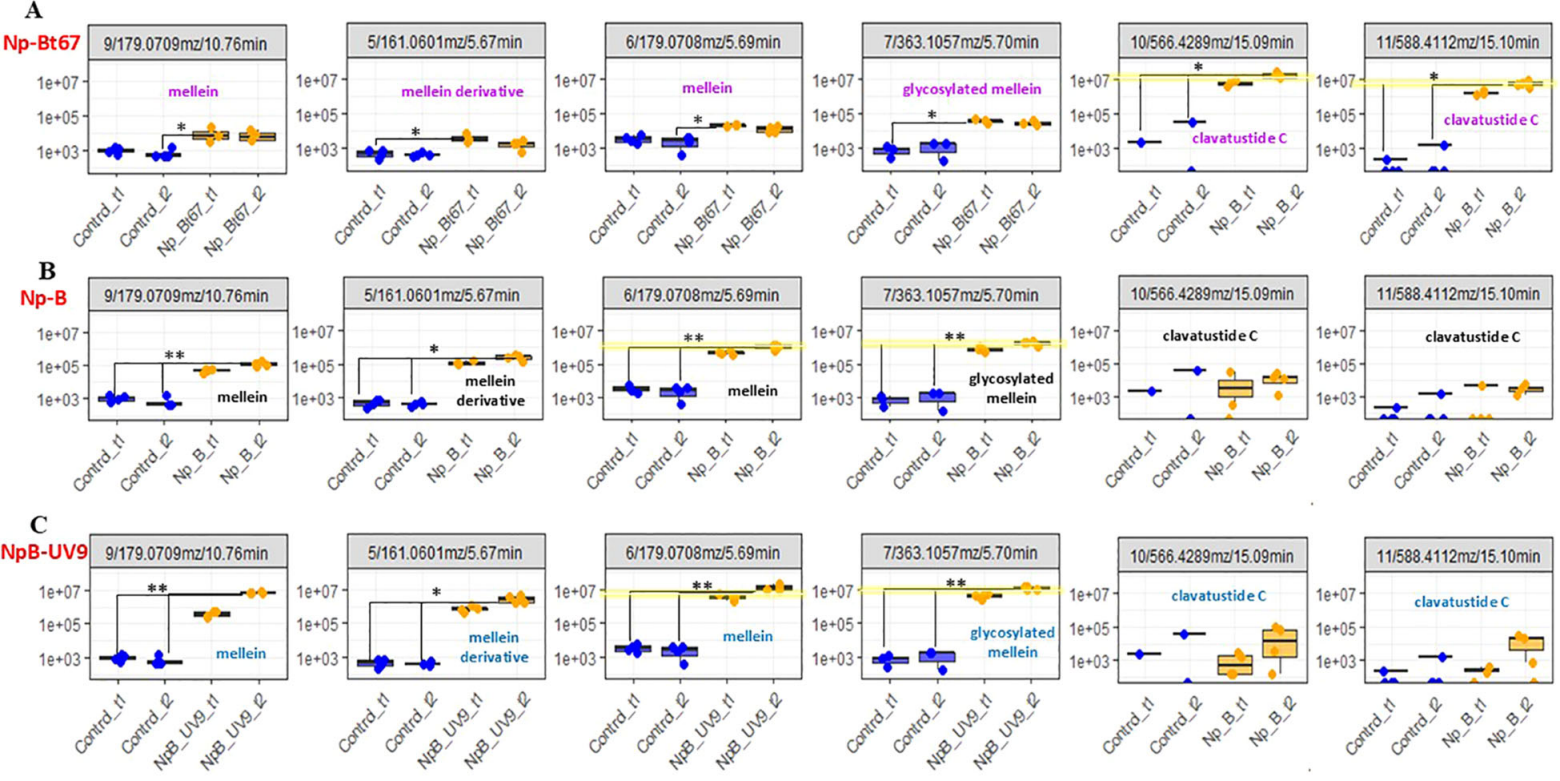
Boxplots showing the variation in LC-MS of the intensity of (*R*)-mellein and clavastustide C into plantlets infected with the *N. parvum* strain Np-Bt67 **(A)**, Np-B **(B)** or NpB-UV9 **(C)** or not (Control), both at 8 dpi (t1) and 14 dpi (t2). Significant differences have been verified by ANOVA testing. The Kruskal–Wallis test reveals significant differences for (*R*)-mellein. *Post hoc* pairwise comparisons have been performed using Dunn’s test with Bonferroni correction, and significant differences are indicated by asterisks (*p < 0.05, **p < 0.01, ***p < 0.001).

**Scheme 1:**
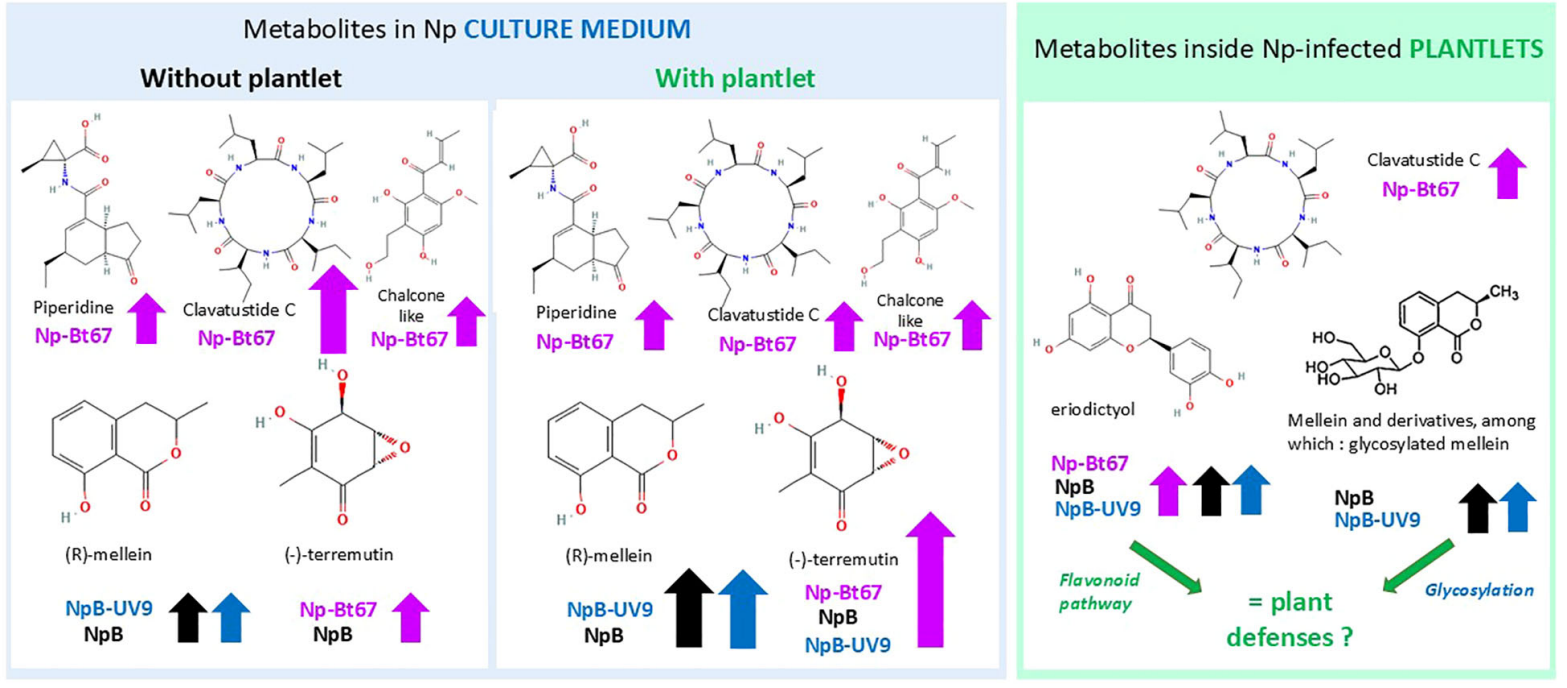
Summary of the metabolomic findings using the sterilized model of plantlets infected by different Np strains (i.e. Np-Bt67, Np-B or NpB-UV9) to better highlight the metabolome incidence of living grapevine on the aggressive metabolome of Np.

Three groups of metabolites discriminate in the comparative analyses based on time (**Figure 10**) and based on the Np strains (**Figure 12**): (*R*)-mellein and derivatives, the cyclopeptide clavatustide C, and the flavonone eridictyol (**Scheme 1**). Since the flavonone exists in Np-infected plantlets whatever the Np strain, only (*R*)-mellein and derivatives, as well as clavatustide have been retained as aggressive molecules to apprehend their differential level *in-planta* by LC-MS (**Figure 13**).

A Kruskal–Wallis test was performed to assess the statistical significance of the differences observed between conditions. *Post hoc* pairwise comparisons were conducted using Dunn’s test with Bonferroni correction, to consider the joint influence of the isolate and post-infection time.

The differences in (*R*)-mellein intensity, and metabolites from this group, between control and infected plantlets are statistically significant for all three Np strains (**Figures 13A–C**), although the intensity detected in Np-Bt67 is relatively low (**Figure 13A**). The highest abundance of (*R*)-mellein is observed in samples infected by NpB-UV9 (**Figure 13C**). However, the difference in production between 8 and 14-dpi is not significant.

To contrast, the differences in clavatustide C intensity is only significant in plantlets infected with Np-Bt67 (**Figure 13A**).

These data confirm the joint influence of grapevine and of the Np strains in the number of metabolites that discriminate in Np-infected plantlets.

### 3.4 HPLC-UV analyses to track (*R*)-mellein and (*-*)-terremutin in both Np-infected culture medium and plantlets

**Figure 14:**
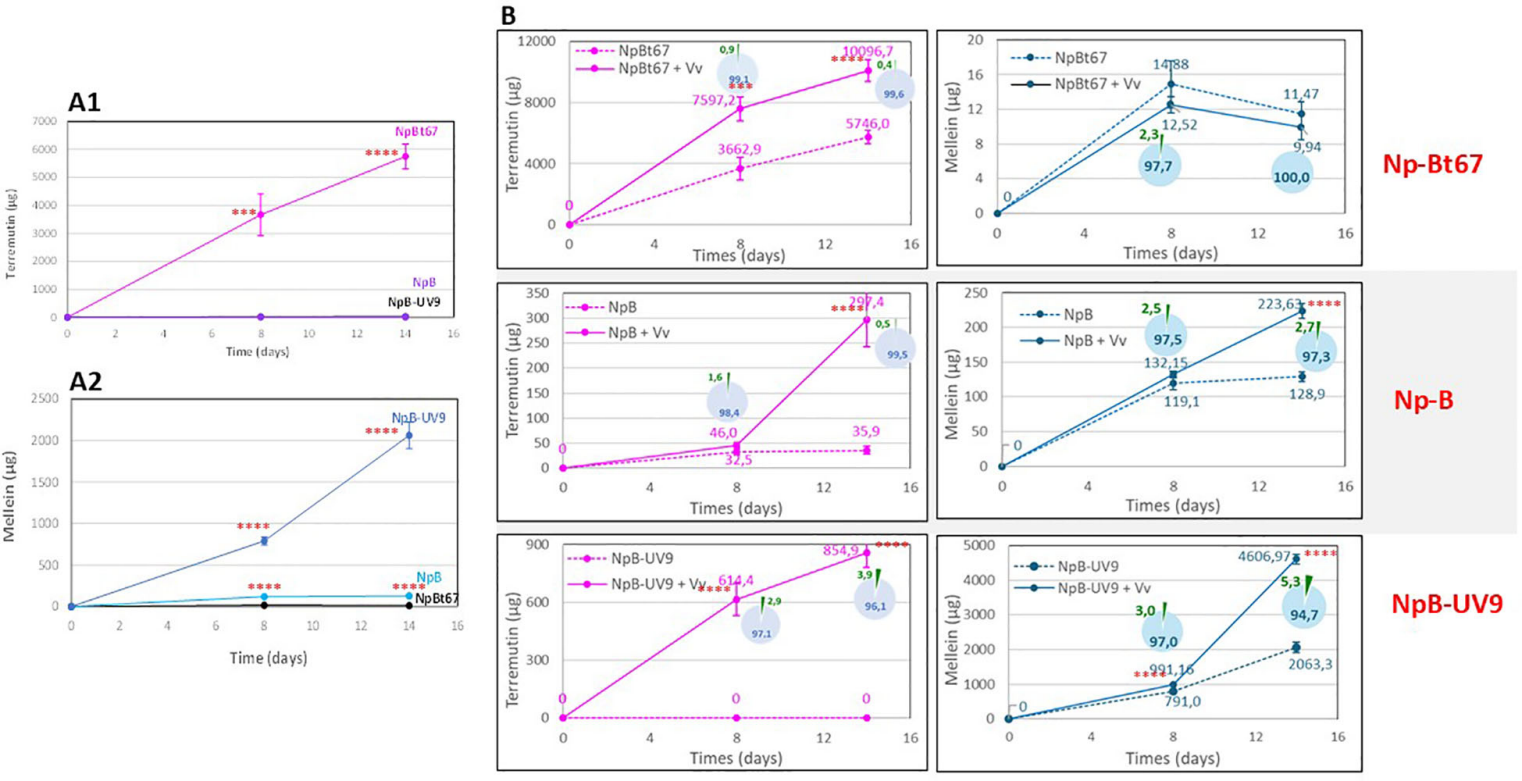
HPLC-UV quantification of (*-*)-terremutin **(A1)** and (*R*)-mellein **(A2)** in the culture medium of the Np strains Np-Bt67, Np-B and NpB-UV9 without the presence of plantlet **(A)**. Similarly, (*-*)-terremutin (B, left side) and (*R*)-mellein (B, right side) have quantified both in the culture medium of the same Np strains with the presence of plantlet (B, solid lines) compared to the same conditions without plantlet (B, dotted lines). The pie chart slices represent the percentage of each phytotoxin quantified either in the culture medium (blue) or in the grapevine tissues (green). A z-test was applied to compare two conditions. (*p < 0.05, **p < 0.01, ***p < 0.001, ****p < 0.0001).

As shown in **Schema 1**, (*R*)-mellein and (*-*)-terremutin production appears as the most affected when a living grapevine is associated to Np culture medium. These two metabolites have therefore been more precisely quantified by HPLC-UV, and immediately at the end of the experiment, both in Np culture medium (that include Np) and in the plantlets (**Figure 14**).

As shown in **Figure 14A1** using the MS-medium, Np-Bt67 produces significantly higher amounts of (*-*)-terremutin (i.e. up to 5746 µg at 14 dpi) than NpB and NpB-UV9, which produce very little (i.e. 35 and 0 µg, respectively). At 14 dpi, production by Np-Bt67 is even significantly higher than that observed at 8 dpi (i.e. 3662 µg) in the same culture medium.

The presence of a living grapevine plantlet stimulates the production of (*-*)-terremutin by all the Np strains (**Figure 14B**, left side), reaching its highest amount with Np-Bt67 (i.e. up to 10096 µg at 14 dpi) compared to Np-B or NpB-UV9 (i.e. up to 297 or 854 µg, respectively). With Np-Bt67, the quantity of (*-*)-terremutin doubles between 8 dpi and 14 dpi when the living plantlet is present (i.e. from 5797 to 10096 µg, respectively). To contrast, with Np-B, the plantlet induces a slight increase of (*-*)-terremutin at 8 dpi (from 32 µg without plantlet to 46 µg with plantlet) that has been amplified at 14 dpi (from 35.9 without plantlet to 297.4 µg with plantlet).With NpB-UV9, the plantlet presence induces a marked increase of (*-*)-terremutin, both at 8 dpi (i.e. 0 to 614 µg) and 14 dpi (i.e. 0 to 855 µg).

The most important part of (*-*)-terremutin remains in the culture medium (i.e. 96.6 to 99.6% of the production) and does not enter inside the plantlet tissues (i.e. 0.4 to 3.6%).

As shown in **Figure 14A2** using the MS-medium, NpB-UV9 is the strain that produces the most of (*R*)-mellein (i.e. 2063 µg). This production also increases significantly between 8 and 14 dpi (i.e. 791 to 2063 µg) in the culture medium.

The presence of a living grapevine plantlet had no significant effect on the (*R*)-mellein production by strain Np-Bt67 (**Figure 14B**, right side). In contrast, a living grapevine plantlet stimulates the (*R*)-mellein production by Np-B and even mire by NpB-UV9, particularly at 14 dpi (i.e. 128 to 223 µg and 2063 to 4606µg, respectively).

As with the other phytotoxin, the most important part of (*R*)-mellein remains in the culture medium (i.e. 94.7 to 100% of the production) despite it can enter in the plantlet tissues (i.e. 0 to 5.3%).

Overall, the HPLC-UV quantification of the two phytotoxins (*-*)-terremutin and (*R*)-mellein confirms the trends observed during HPLC-MS/MS analyses, validating the consistency between the two analytical approaches.

## 4 Discussion

To date, no sustainable control method is sufficiently effective to protect grapevine to GTDs in vineyards, especially against Botryosphaeria dieback (BD) due to *N. parvum* (Np) (Fontaine et al., 2025; Leal et al., 2024; Mesguida et al., 2023; Mondello et al., 2018). Additionally, the current context of climate change is risky for plants and related beneficial microorganisms. It is therefore essential to develop, in addition to prophylactic methods, new protection products that weaken the pathogen aggressiveness in particular by targeting its virulence factors. To achieve this, it is essential to be sure of what drives Np virulence and how it is exacerbated. To date, mobile phytotoxic metabolites are described as virulent factors clearly contributing to Np pathogenicity (Trotel-Aziz et al., 2019, 2022), and transformant constructions are in progress to establish the clear causal link between the production of some virulent factors and BD expression (Trotel-Aziz et al., 2022). In parallel, the development of a simple and highly reproducible plantlet model will enable to quickly track and confirm the whole virulence activity of Np, with possible ways of detoxication, to later develop the best strategy to control BD. One of the objectives is therefore to track the whole metabolomes of distinct Np strains, described as more or less aggressive *in-planta*. Another objective is to evaluate the incidence of a living host on the virulence activity of *N. parvum* strains (Np). To achieve these aims, this work takes advantage of a sterilized model of grapevine plantlets (*Vitis vinifera* L. cv. Chardonnay). Virulence is assessed by the percentage of necrosis in infected plantlets in response to Np strains whose metabolome is analyzed. This study is therefore the first reporting on both: (i) the whole metabolome of distinct Np strains (i.e. Np-Bt67, Np-B and NpB-UV9) growing in a sterilized agar-modified Murashige-Skoog (MS) medium, and (ii) the incidence of living grapevine plantlet on this metabolome, among which virulence factors. Metabolites are analyzed in both culture medium and plantlet tissues by LC-MS, with an additional HPLC analysis for the major phytotoxins mellein (M) and terremutin (T).

The virulence of the Np strains has been assessed by the percentage of necrosis in infected plantlets in response to Np strains. As already reported with cuttings, Np-Bt67 exhibits the highest level of aggressiveness compared to Np-B and NpB-UV9 (Trotel-Aziz et al., 2022). Other studies also report on the very aggressive potential of Np-Bt67 (Abou-Mansour et al., 2015; Bellée et al., 2017). This Np strain is described to overproduces (*-*)-terremutin (T) but almost no (*R*)-mellein (M) *in-vitro* (Abou-Mansour et al., 2015; Bellée et al., 2017; Trotel-Aziz et al., 2022).

The metabolome of each Np strain, grown in a carbohydrates rich culture medium, has been analyzed (**Schema 1**). Some of the clusters of glycosylated compounds and saccharides annotated in the molecular network of this study could originate from the culture medium. These compounds have potentially fueled Np metabolism, as this might also occur under nutrient-rich natural conditions.

Analysis of the metabolome of Np-Bt67, together with additional target HPLC analysis, reveals that this most aggressive strain produces the highest amount of (-)-terremutin (T) in this rich culture medium (i.e. 5746 µg at 14 dpi), while mellein (M) remains low (i.e. up to 14 µg). These data are consistent with those of Abou-Mansour et al. (2015); Andolfi et al. (2011); Djoukeng et al. (2009) and Trotel-Aziz et al. (2022) using different bioassays. Additionally, this study shows an early and elevated T-production by Np-Bt67, in good concordance with its strong aggressiveness toward plantlets, suggesting that T could play a more decisive role than M in Np virulence (Abou-Mansour et al., 2015; Andolfi et al., 2011; Djoukeng et al., 2009; Trotel-Aziz et al., 2022). Other putative metabolites are also detected in Np-Bt67 metabolome by UHPLC-MS/MS, among which cyclopeptides (Clavatustide C, Luminmide B), piperidine compounds, steroid esters, and chalcone-like polyketides. Clavatustide C is a cyclic peptide previously described as a metabolite involved in abiotic stress response of fungi such as *Aspergillus clavatus* (Quiñonero et al., 2024; Ye et al., 2014). Piperidine is described as a fungal alkaloids with antimicrobial and phytotoxic properties (Phainuphong et al., 2018; Thawabteh et al., 2024; Zhang et al., 2022). A high production of these compounds could therefore offer a competitive advantage to Np-Bt67 toward other microorganisms. Steroid esters are known as key components of fungal lipid metabolism, primarily serving as storage forms of steroids within lipid droplets (Czabany et al., 2007). Among them are different subclasses such as sterols that participate in membrane organization and signaling at the plant-microbe interface, affecting compatibility and pathogenicity outcomes (Der et al., 2024). Changes in sterol biosynthesis would notably affect fungal virulence‐related traits (Derbyshire et al., 2015). Despite chalcones are classically considered plant metabolites, recent studies have demonstrated that fungi have evolved unique biosynthetic mechanisms to produce metabolites structurally analogous to plant-defense compounds, including the direct biosynthesis of chalcones by fungal polyketide synthase (PKS) systems (Furumura et al., 2023). This highlights the potential role of such flavonoids in fungal ecology, including microbial competition or pathogenicity.

In the less aggressive Np-B and NpB-UV9 strains, a glycoside compound discriminates (i.e. a nitrogen-containing glycosylated compound). Glycosylation is described to increase solubility and allow the storage and transport of small molecules (Bowles et al., 2005; Jones and Vogt, 2001). Glycosylation is also useful as a self-protection mechanism used by fungi and plants to detoxify toxins (Tian et al., 2016), but also to hide toxins that will later develop the full aggressiveness of the pathogen (Stranska et al., 2026). Consistently, weak amounts of toxins are produced by Np-B and NpB-UV9.

The less aggressive Np-B strain exhibits weak amounts of both T and M *in-vitro* (i.e. up to 35 µg and up to 128 µg, respectively at 14 dpi), while the mid-aggressive NpB-UV9 displays the strongest M amount (i.e. 2063 µg at 14 dpi), but no T on the MS medium. Similar trends of M and T were described by Trotel-Aziz et al. (2022) *in-vitro*, with smaller phytotoxin quantities on PDA plates compared to the MS medium. Although both M and T have been described to repress grapevine defenses in favor of disease expression (Trotel-Aziz et al., 2019), the highly aggressive Np strain Np-Bt67 *in-planta* remains the only one to overproduce T *in-vitro* (i.e. 5746 µg at 14 dpi). One can therefore wonder whether the amount of toxin produced *in-vitro* really predisposes to the amount that will produce Np when exposed to a living plant, and similarly for the entire Np metabolome. Therefore, additional experiments have been performed to observe the plantlet effect on the virulence activity of Np among which its capacity to produce each toxin.

Therefore, each strain exhibits a specific metabolic signature regarding their aggressive molecules: Np-Bt67 overproduces T, Np-B produces weak amounts of T and M, while NpB-UV9 overproduces M, as described by Trotel-Aziz et al. (2022) when Np is growing onto a MS medium (**Schema 1**).

When exposed to a living grapevine plantlet, the metabolome of each Np strains change (**Schema 1**). Notably, the production of one metabolite is amplified by all the Np strains in response to plantlet presence: (*-*)-terremutin (T), confirming its role as a central phytotoxin as highlighted by Abou-Mansour et al. (2015); Andolfi et al. (2011); Djoukeng et al. (2009); Bellée et al. (2017) and Trotel-Aziz et al. (2022).

Np-Bt67 still exhibits the highest amount of T, with a pronounced amplification of T with plantlet presence (i.e. up to 10096 µg at 14 dpi versus 5746 µg without plantlets). In contrast the content of (*R*)-mellein (M) remains low. No more increase in the production of other metabolites appears in response to the plantlet presence, despite the same discriminating metabolites are present in the metabolome of Np-Bt67 with or without plantlet: the cyclopeptides clavatustide C and luminmide B, piperidines, steroid esters and chalcone-like PK. Therefore, their productions would not serve Np aggressiveness against grapevine. However, as reported in fungi, bacteria, plants and algae, among which marine organisms (Dalsgaard et al., 2004; Sobolevskaya et al., 2005; Wang et al., 2018), the cyclic peptides (i.e. clavatustide C and Luminmide B) could serve as reliable markers of fungal presence or infection.

The production of T by Np-B is also amplified in response to the plantlet presence (up to 297 µg, instead of 35 µg at 14 dpi without plantlet), as well as M that reaches 223 µg *versus* 128 µg at 14 dpi with plantlet. The weakly aggressive Np-B therefore produces both toxins in the presence of plantlet, but in their weakest amount compared to Np-Bt67 for T (up to 10096 µg at 14 dpi) or to NpB-UV9 for M (up to 4606 µg at 14 dpi). Once again, there is a good correlation between the strong or weak Np-aggressiveness toward plantlets (i.e. plant phenotypes, **Figure 1**) and the strong or weak production of phytotoxins. This still reinforces the idea of ​​a decisive role for phytotoxins *-especially T-* in the virulence of Np. Indeed, even the mid-aggressive NpB-UV9 that did not produce T without the presence of plantlet, starts to produce T with the plantlet presence (i.e. 854 µg at 14 dpi), therefore in higher quantity than Np-B. NpB-UV9 also displays a strong M production upon the plantlet challenge (i.e. 4606 µg at 14 dpi, *versus* 2063 µg without plantlet). But the highly aggressive nature of Np-Bt67 toward plantlet is independent of M accumulation (since it produces almost no M).

Interestingly, while no more stimulation in the production of other metabolites appears in response to Np-B exposure to plantlet, glycosides production is increased in response to NpB-UV9 exposure to plantlet. The same discriminating metabolites are also detected in the metabolome of the less aggressive Np-B, with or without plantlet (i.e. a nitrogen-containing glycosylated compound). However, similar glycosides do not discriminate while analyzing the metabolome of the most aggressive Np-Bt67. These data therefore suggest that these glycosides do not contribute to Np virulence by T, but possibly the NpB-UV9’s self-protection or that of grapevine when associate to NpB-UV9 culture medium (Stránská et al., 2026; Thuan et al., 2024; Tian et al., 2016). Regarding glycoside metabolites, it should be reminded that their identification relies on MS/MS analyses and SIRIUS predictions, and therefore remains putative. Currently, their biological activity has not yet been tested.

Whatever, one can retain that there is a common feature to the Np strains (i.e. Np-Bt67, Np-B and NpB-UV9) when they are co-cultivated with a living grapevine plantlet: a marked stimulation of T production by all the Np strains (**Schema 1**).

Looking now at the metabolome of grapevine plantlets, marked differences appear between control and infected samples. Several metabolites, including amino acid derivatives such as pyroglutamic acid, betaine and chlorophyll-related compounds (i.e. methyl pheophorbide), are more abundant in control plantlets and decrease during infection. This pattern may reflect metabolic reprogramming and tissue degradation associated with Np infection (Cheaib and Killiny, 2025; Tanaka and Ito, 2025).

Other metabolites over-accumulate in infected plantlets (**Schema 1**), highlighting active grapevine–Np interactions. Among them, the flavanone (or chalcone) eriodictyol is detected in all the Np-infected plantlets and accumulated progressively, reaching a maximum at 14 dpi. Eriodictyol is a grapevine PKS-derived flavonoid involved in defense against fungal attack (Escobar-Avello et al., 2019; Rusjan & Mikulic-Petkovsek, 2015). Several flavonoids, including eriodictyol, have also been reported to inhibit PKS activities by interfering with substrate binding (Hinderer and Seitz, 1985; Tiwari and Shankar, 2018). This plant metabolite could therefore be useful to inhibit the fungal PKS responsible for T and M biosynthesis. Eriodictyol accumulation over-time also suggests a sustained activation of the phenylpropanoid pathways in response to fungal infection, possibly due to increasing tissue damages and resulting DAMPs (damages associated molecular patterns) acting as elicitors of plant defenses (Jasiński et al., 2009; Päsold et al., 2010; Ramaroson et al., 2022).

In addition to host-derived metabolites, several fungal compounds are detected inside the plant tissues (**Schema 1**). Among them, the cyclopeptide Clavatustide C is specific to plantlet exposure to Np-Bt67, but also M and derivatives when plantlets are exposed to Np-B and NpB-UV9. These data therefore clearly indicate that certain fungal metabolites can penetrate and accumulate in grapevine, possibly as a detoxified form. Indeed, glycosylated forms of M are detected inside the plantlet tissues in response to Np-B or NpB-UV9. As discriminating metabolites, this suggests that grapevine actively detoxifies M by conjugation to carbohydrates. Glycosylation is a well-known plant defense mechanism to neutralize toxic molecules such as secreted secondary metabolites produced by phytopathogen (Gachon et al., 2005; Le Roy et al., 2016; Pedras et al., 2001). The carbohydrate-rich culture medium of plantlets could therefore favor their detoxifying metabolism, as well as the other nutrients from this rich culture medium.

In contrast, no conjugate of T appears inside the plantlets. This suggests that grapevine could selectively detoxify some of the fungal metabolites entering its tissues, thus partially limiting Np toxicity. Maybe grapevine does not yet possess the right enzymatic system to detoxify T, thus justifying an increased sensitivity to Np-Bt67 that highly produces T. Consistently, T does not appear as a discriminant metabolite entering the plantlet tissues to contrast with clavatustide, eriodictyol, and M with derivatives.

Finally and importantly, both T and M remain largely outside the plantlet tissues (i.e. 96.6 to 99.6% for T, and 94.7 to 100% for M). This is further evidence of the probable decisive role of phytotoxins in the virulence of Np toward living grapevine.

Whatever, and despite no plantlet defenses appear directly to combat T production by Np-Bt67, a common plantlet answer to all the Np strains is flavonoid accumulation (i.e. the chalcone eriodictyol), as well as M glycosylation when facing the less aggressive Np-B and NpB-UV9. In order to better investigate the reciprocal interaction of grapevine (i.e. detoxification) with Np (i.e. M, T, or their biosynthesis enzymes), it would be useful to combine single-cell/nucleus and spatial transcriptomics (Ali et al., 2026) to decipher the coordinated defenses/adaptations of the different types of plant cells and tissues against Np.

## 5 Conclusion

Overall, these results highlight an early and significant production of (*-*)-terremutin by Np-Bt67, the highly aggressive *N. parvum* strain compared to Np-B and NpB-UV9. (*-*)-Terremutin production by Np strains is greatly amplified in the presence of a living vine plantlet regardless of the Np strain, therefore even with the NpB-UV9 strain which does not initially produce (*-*)-terremutin. In addition, plant tissue analysis shows that infected grapevine plantlets activate defense-related pathways such as flavonoid biosynthesis and glycosylation processes, likely involved in the inhibition of production of both toxins and the neutralization of bioactive compounds such as (*R*)-mellein. The review by Ali et al. (2025) further reports on the signaling context upstream of plant immune activation, particularly on the roles of ROS/RNS in pathogen-triggered flavonoid induction.

Given that the grapevine’s defenses are ineffective against Np-Bt67 which overproduces (*-*)-terremutin, and that grapevine even triggers the overproduction of (*-*)-terremutin regardless of the Np strain, it is suggested that plant-made substrates feed more the Np-PKS to promote virulence, than the Vv-PKS to promote grapevine’s defenses. Complementary transcriptomic approaches could also enable the search for molecular signals from living vines capable of specifically triggering an overproduction of (*-*)-terremutin in Np.

Despite the use of different extraction methods could potentially allow to identify other plant metabolites in the near future, this work provides an interesting view on Np virulence strategy against grapevine and on host defense responses during Np infection, both involving polyphenols and carbohydrate pathways. This work also highlights several metabolites as promising biomarkers for early detection and monitoring of fungal infections. Among them, cyclopeptides (i.e. clavatustide C and luminmide B) appear as potential new biomarkers for the presence of Np. In particular, the presence of clavatustide C inside infected plantlets makes it a promising new biomarker for early infection of grapevines by the highly aggressive strain of Np.

## Data Availability

The original contributions presented in the study are included in the article/**Supplementary Material**. Further inquiries can be directed to the corresponding author.
